# Radiolabeled Nanogels: From Multimodality Imaging to Combination Therapy of Cancer

**DOI:** 10.1002/smsc.202400298

**Published:** 2025-06-19

**Authors:** Sanchita Ghosh, Weibo Cai, Rubel Chakravarty

**Affiliations:** ^1^ Radiopharmaceuticals Division Bhabha Atomic Research Centre Trombay Mumbai 400085 India; ^2^ Homi Bhabha National Institute Anushaktinagar Mumbai 400094 India; ^3^ Departments of Radiology and Medical Physics University of Wisconsin‐Madison Madison WI 53705 USA

**Keywords:** cancer, combination therapy, drug delivery, multimodality imaging, nanogel

## Abstract

Advancements in nanotechnology over the past few decades have offered tremendous possibilities toward cancer theranostics. Radiolabeled nanogels (NGs) represent a promising nanoplatform in this direction, offering a multifunctional toolset for both imaging and therapeutic interventions. This review encapsulates the progressions and potential of radiolabeled NGs in the realm of cancer research. Firstly, multifunctional radiolabeled NGs serve as potent contrast agents for multimodality imaging, enabling precise visualization of tumor sites through various techniques such as positron emission tomography, single‐photon emission computed tomography, optical imaging and magnetic resonance imaging. Furthermore, by incorporating more than one therapeutic payload such as chemotherapeutic drugs, nucleic acids, and/or therapeutic radionuclides, they enable synergistic treatment modalities that address the heterogeneity of cancer cells and their microenvironment. This combination approach allows for enhanced therapeutic efficacy while minimizing systemic toxicity, addressing challenges associated with conventional cancer therapies. Furthermore, the radiolabeling of NGs provides a means for real‐time monitoring of therapeutic distribution and pharmacokinetics, offering valuable insights into treatment response and optimization. Overall, radiolabeled NGs represent a promising platform for the integration of multimodality imaging and combination therapy in the fight against cancer with increased efficacy, reduced toxicity, and improved patient outcomes.

## Graphical abstract

Nanogel refers to nanosized particles formed by physical or chemical crosslinking of polymers. Rational design and refinement of nanogel properties are imperative to unlock their full potential, ensuring tailored solutions for personalized cancer management. This review elucidates the transformative potential of radiolabeled nanogels in revolutionizing cancer diagnosis and therapy, paving the way for the next generation “precision medicine” approaches.

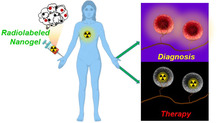

## 1 Introduction

Despite tremendous progress in medical science, cancer is still one of the leading causes of morbidity and mortality worldwide. According to the GLOBOCAN 2024 report, nearly 20 million new cancer cases were reported in the year 2022 alone and more than 9.7 million people died due to the disease.^[^ ^1^ ^]^ Throughout the world, there has been tremendous investment toward research for early diagnosis and treatment of cancer and in this direction nanotechnology or nanomedicine has taken giant strides in recent years.^[^ ^2^, ^3^, ^4^ ^]^ Although there are certain limitations and controversies associated with nanomedicine, it still offers the hope for efficient diagnosis with more accuracy and reduced toxicity than conventional cancer treatment methodologies.^[^ ^5^, ^6^ ^]^ Moreover, novel nanoplatforms offer various diagnosis and therapy possibilities in a single nanosystem. It would not be an exaggeration to state that nanotechnology in the near future will play an important role in early detection of cancer, drug delivery including image‐guided drug delivery, targeted therapy, and monitoring therapeutic progress post‐treatment.^[^ ^7^ ^]^ Therefore, development in nanomedicine can potentially lay the foundation stone of modern‐day personalized cancer management.^[^ ^8^, ^9^, ^10^, ^11^ ^]^

Taking into consideration different kinds of cancers, individual patients require different theranostic nanoformulations. Currently, magnetic fields, ultrasound, optical agents, and a variety of radiation sources are used as the basis for imaging techniques for diagnosis.^[^ ^12^, ^13^, ^14^ ^]^ These techniques include magnetic resonance imaging (MRI), ultrasonography (US), computed tomography (CT), single‐photon emission computed tomography (SPECT), and positron emission tomography (PET). To enhance the quality of images, various nanoparticle‐based contrast agents, such as iron oxide, gadolinium have been used for MRI.^[^ ^15^, ^16^ ^]^ For SPECT or PET imaging technique, nanoparticles radiolabeled with the appropriate diagnostic radionuclides are being utilized.^[^ ^17^, ^18^, ^19^, ^20^ ^]^ Several light‐based diagnostic techniques such as photoacoustic imaging, fluorescent imaging, and photothermal imaging technique are also becoming research hot spots.^[^ ^21^, ^22^, ^23^, ^24^ ^]^ Aside from diagnosis, light can also trigger therapy, for instance, in photodynamic therapy singlet oxygen can induce cancer cell death and in photothermal therapy heat is generated to kill cancer cells.^[^ ^25^, ^26^, ^27^ ^]^ The incorporation of therapeutic radionuclides in nanosystems additionally augment their tumor treatment capability, leveraging the distinct pharmacokinetic characteristics of “nano‐radiopharmaceuticals”.^[^ ^18^, ^28^, ^29^ ^]^ Although most of the research on nanomedicine is laboratory based, scientific communities all over the world have initiated their translation to the clinics for the benefit of cancer patients.^[^ ^30^, ^31^ ^]^

Over the last several years, numerous nanoplatforms have been developed for cancer management, which are based on metallic nanoparticles, dendrimers, micelles, lipid nanoparticles, quantum dots, carbon nanotubes, nanogels (NGs), etc.^[^ ^32^, ^33^, ^34^, ^35^, ^36^, ^37^, ^38^, ^39^ ^]^ Despite excellent attributes, some micelles and lipid nanoparticles still lack in stability compromising their efficacy in vivo.^[^ ^40^, ^41^ ^]^ Additionally, quantum dots and metallic nanoparticles might impart cytotoxicity concerns due to the oxidative stress generated by hazardous metals.^[^ ^42^, ^43^ ^]^ On the contrary, carbon nanotubes and dendrimers show drawbacks in terms of low solubility in aqueous medium and induce nonspecific toxicity in certain cases.^[^ ^44^, ^45^ ^]^ In fact, the biosafety of most of the inorganic nanoplatforms remains a contentious topic because of their potential toxicity and limited biodegradability in medical settings.^[^ ^46^, ^47^ ^]^ Several additional factors such as their size, shape, surface functional groups, and dose‐dependent characteristics might also contribute toward toxicity in healthy human cells, tissues, and organs. Nevertheless, there are few inorganic nanoparticles, like calcium phosphate, silica, and gold nanoparticles, which are potentially nontoxic and hold significant promise toward clinical translation.^[^ ^48^, ^49^ ^]^ Overall, there is a significant challenge in developing new nanomaterials that combine safety with high performance.^[^ ^50^ ^]^ Recently, NG, a 3D‐crosslinked polymeric material, got the attention of the researchers as its size and properties can be tuned easily.^[^ ^51^, ^52^, ^53^ ^]^ NGs refer to nanosized particles formed by physical or chemical crosslinking of polymers.^[^ ^54^, ^55^ ^]^ The diverse applications of NGs cover from food industry, agriculture to fabrication of organic materials.^[^ ^56^, ^57^, ^58^ ^]^ Nevertheless, major applications of NGs are in biomedical area, which comprises drug delivery, vaccine fabrication, tissue engineering, and cancer therapy.^[^ ^59^, ^60^, ^61^, ^62^ ^]^ Due to the unique structure and properties of NGs, they are considered superior to several conventional nanoformulations. For instance, NGs are highly biocompatible and behave like natural tissues due to their high water content, possess high surface area contributing toward high loading capacity for drug, DNA/RNA sequence, imaging agent, and so on.^[^ ^63^, ^64^ ^]^ The Hamaker constant of NGs is comparable to that of water, which contributes to their excellent stability in biological fluids due to minimal driving forces for their aggregation.^[^ ^65^ ^]^ Additionally, the flexibility and softness of NGs may facilitate their easier penetration through human skin while preserving the therapeutic activity, in contrast to more rigid nanoparticles.^[^ ^66^ ^]^ The flexible nature of NGs also helps extend their presence in the bloodstream by minimizing the likelihood of being trapped by macrophages.^[^ ^67^ ^]^

As an arsenal of monomers is available for the synthesis of NG, multifunctional and biocompatible polymer chains can be chosen to synthesize highly functionalized, less toxic NGs for biomedical applications. So far, ten‐to‐few hundred nanometer‐sized NGs have been studied for targeted drug delivery as NGs serve as an ideal transport system in living objects.^[^ ^55^ ^]^ Stimuli‐responsive NGs have a greater significance in cancer research because of their ability to modify their physicochemical characteristics such as drug loading capacity, hydrophobicity, and permeability in the internal network in response to external stimuli, such as changes in pH, light, magnetic fields, temperature, and volume. In general, payloads of non‐responsive NGs are uncontrollably released by diffusion, which can be controlled by conjugating them with imaging and therapeutic agents by cleavable groups. On the contrary, the physicochemical behavior of the responsive NGs can be controlled by external stimulation like pH, temperature, ionic strength of the medium, radiation, enzymatic activity, magnetic stimulation, and many more.^[^ ^68^ ^]^ Regardless of the excellent properties of NGs, it is desirable to monitor the biodistribution, pharmacokinetics, and pharmacodynamics properties after in vivo administration.^[^ ^69^, ^70^, ^71^ ^]^ In this direction, molecular imaging techniques such as MRI, US, optical imaging, PET, and SPECT provide insights about the in vivo behavior of the NG. Particularly the nuclear techniques (SPECT and PET) allow quantifying the pharmacokinetics data and the release protocol of the entrapped compounds from radiolabeled NGs.^[^ ^72^, ^73^, ^74^, ^75^ ^]^

**Figure 1:**
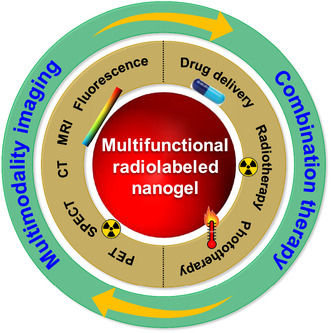
Schematic representation of multifunctional radiolabeled NGs for use in multimodality imaging and combination therapy for advanced cancer care.

Radiolabeling of NGs has emerged as a pivotal technique, fostering extensive research into understanding the intricate fate of NGs both in vitro and in vivo.^[^ ^73^ ^]^ This review underscores the transformative impact of radiolabeling on enhancing the diagnostic and therapeutic efficacy in cancer management. Radiolabeled NGs offer unparalleled opportunities for non‐invasive PET and SPECT imaging, thereby enabling precise tumor localization and monitoring of therapeutic responses in real‐time. Additionally, combining different imaging modalities such as MRI, CT, optical imaging with PET and SPECT allows a more detailed and accurate understanding of structural and functional information about cancerous lesions (**Figure** **1**). On the contrary, the combination of radiolabeling with therapeutic payloads empowers NGs as a multifunctional platform for targeted drug delivery, enhancing therapeutic outcomes while minimizing off‐target effects (Figure 1). Through a comprehensive analysis of recent advancements, this review elucidates the transformative potential of radiolabeled NGs in revolutionizing cancer diagnosis and therapy, paving the way for the next generation of precision medicine approaches.

## 2 Synthesis of NGs

**Figure 2:**
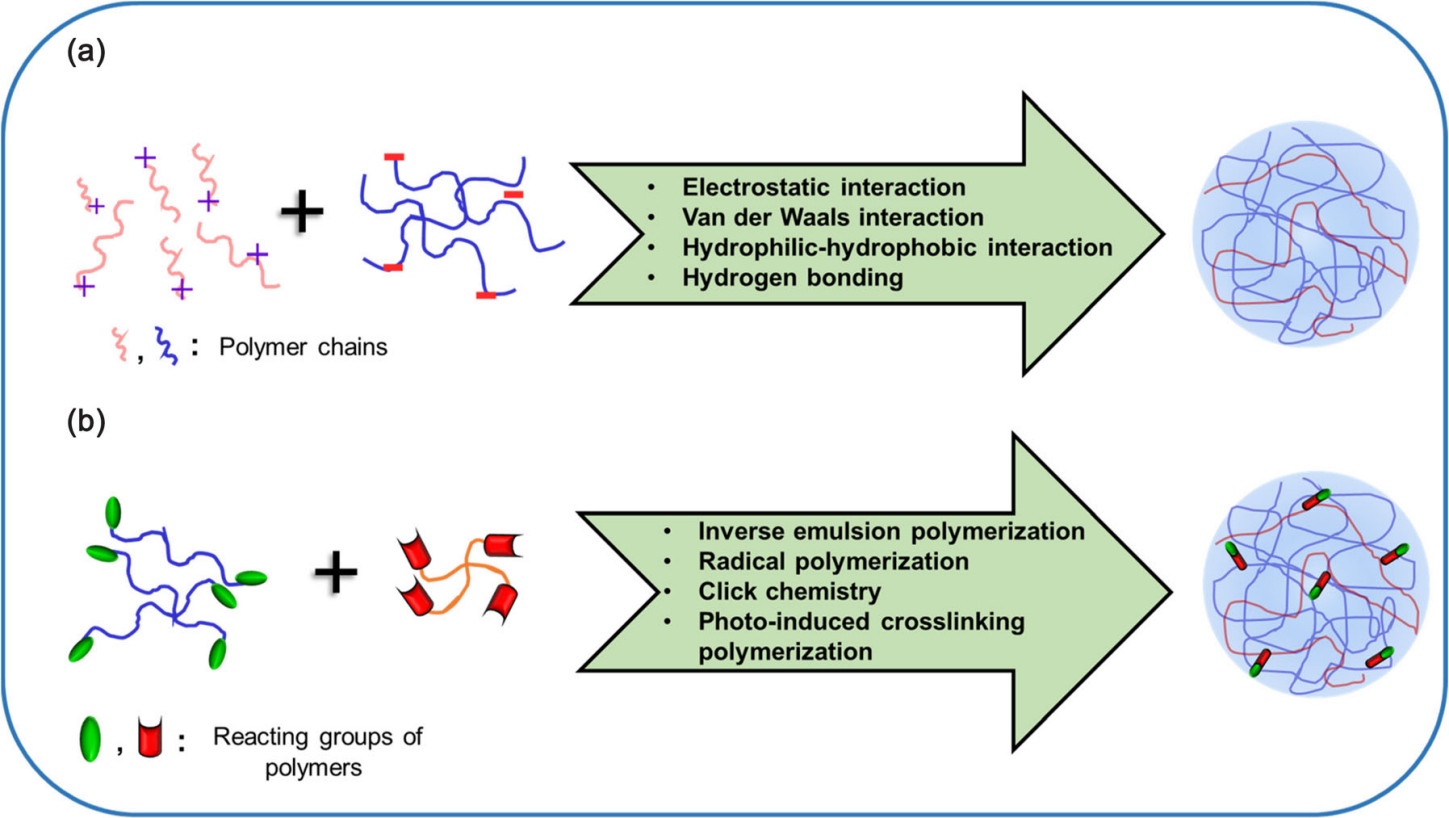
Illustration of different crosslinking methods for synthesis of NGs. a) physical crosslinking method and b) chemical crosslinking method.

Numerous techniques have been utilized for the synthesis of NGs, which can broadly be classified into two categories: 1) chemical crosslinking; and 2) physical crosslinking methods, as illustrated in **Figure** **2**. The chemical crosslinking method imparts more stability compared to the physical one due to covalent bond formation between functional groups present in the polymeric building blocks. On the contrary, physical crosslinking depends on non‐covalent interactions like host–guest interaction, weak Van der Waals force, hydrogen bonding, electrostatic interaction, and hydrophobic–hydrophilic interaction.^[^ ^76^ ^]^ Although a noncovalent bond is weaker than a covalent bond, the physical crosslinking process is more convenient and flexible as complex reaction conditions are not required.^[^ ^77^, ^78^ ^]^ This section briefly summarizes the two procedures for the synthesis of NGs. Some examples of different crosslinking methods are provided in **Table** **1**.

**Table 1:** Different crosslinking methods used for synthesis of NGs.

Type	Serial no.	NG used	Crosslinking method	Size	Stability	Application	References
Physically crosslinked NG	i	Cholesterol‐bearing pullulan‐based NG	Hydrophobic interaction	24.3 nm	Stable in 10% serum medium up to 1 h	Delivery of quantum dots into living cells	[79]
	ii	Chitosan‐based NG	Electrostatic interaction	40–140 nm	Zeta potential −39 to −48 mV	Photodynamic treatment of the inflamed joints	[91]
Chemically crosslinked NG	i	L‐glutamic acid‐based NG	Inverse miniemulsion polymerization and radical polymerization	280–370 nm	Thermally stable up to 200 °C	Hydrophilic drug nanocarriers	[93]
	ii	Poly(vinyl alcohol)‐based NG	Photo‐induced crosslinking polymerization	179.7 ± 1.7 nm	Degrade at acidic pH	Drug delivery	[104]
	iii	Cross‐linked prodrug NG	Azide–alkyne click reaction	60.6 ± 13.7 nm	Stabile in high salt concentration, and long‐time incubation in phosphate‐buffered saline	Intracellular drug delivery	[99]
	iv	N‐isopropylacrylamide‐based NG	Sonochemically induced reversible addition‐fragmentation chain transfer (RAFT) polymerization	33–272 nm	Not reported	Not reported	[94]

### 2.1 Physical Crosslinking Method

Physically crosslinked NGs are formed by weak physical interaction under mild reaction conditions, which circumvents the need for using harmful chemicals and thus induce less toxicity. One typical example of a physical interaction mechanism is the hydrophobic–hydrophilic interaction in which hydrophobic groups are attached to hydrophilic polymeric chains, which self‐assemble to form NGs in an aqueous medium. For instance, Toita et al. grafted N‐isopropylacrylamide (NIPAM) blocks to deliver quantum dots into living cells.^[^ ^79^ ^]^ Another group synthesized NIPAM‐grafted chitosan‐based NGs for drug delivery.^[^ ^80^ ^]^ In both the cases, pullulan was used as the hydrophilic part.^[^ ^81^ ^]^ Although monomeric NIPAM is known to be toxic, polymeric NIPAM did not exhibit cytotoxicity in a few cell lines studied.^[^ ^82^ ^]^ However, it was reported that endothelial cells consistently showed reduced viability toward NIPAM polymer.^[^ ^82^ ^]^ Hence, a detailed study is warranted to assess the toxicity effects. Besides this, lactic acid, acrylics are used as hydrophobic moiety while the hydrophilic moiety comprises of saccharides and polyethylene glycol (PEG) chains.^[^ ^83^, ^84^, ^85^, ^86^ ^]^ Folic acid is another hydrophobic targeting ligand for folate receptor, which is overexpressed in cancers of the breast, lung, ovary, kidney, etc.^[^ ^87^ ^]^ The hydrophobic part of the folic acid can be attached to hyaluronic acid to form NGs for targeting human breast cancer cells.^[^ ^88^ ^]^ However, the stability of these NGs was low because of the physically crosslinked structure. Ionic interaction can happen between negatively charged molecules and positively charged surfactants.^[^ ^89^ ^]^ These NGs have better stability than the former one. For instance, the carboxyl group present in alginate and the amino group present in chitosan form NGs via electrostatic interaction. Due to the cationic nature of chitosan, it can easily form complex with negatively charged DNA. However, very strong interaction can prevent the dissociation of DNA and limit gene transfer efficiency.^[^ ^90^ ^]^ Moreover, multivalent anions or cations can also be used to create NGs via ionotropic gelation. For instance, the anionic tripolyphosphate (TPP) can cross‐link cationic chitosan (CTS), which has a pK_a_ of around ≈6.5, forming NGs through ionic bridging of the CTS chains.^[^ ^91^ ^]^ In supramolecular crosslinked NGs, hydrogen bond formation is more feasible and straightforward between functional groups such as –OH, and −NH_2_. However, the stability of NGs formed by hydrogen bonding network is poor and affected by pH and solvent medium due to the weak nature of this bond. Physically crosslinked NGs are mechanically less stable compared to chemically cross‐linked NGs. Hence, they may degrade easily and produce harmful decrosslinking by‐products during circulation in blood thereby causing pre‐releases of the drug and hence toxicity. These drawbacks limit the use of physically crosslinked NGs in drug delivery applications.

### 2.2 Chemical Crosslinking Method

Chemical crosslinking method is the most deployed one for NG synthesis as it yields a thermodynamically more stable product with high colloidal stability. Although covalent bond formation leads to high stability, it can cause incomplete drug release in certain cases.^[^ ^85^ ^]^ Hence, it is essential to choose proper methods and materials. Various methodologies have been developed for synthesizing chemically crosslinked NGs which are as follows: 1) Inverse emulsion polymerization: This kind of polymerization operates by generating uniform, kinetically stable droplets within a continuous phase. This approach confines the polymerization process to these droplets, influencing the size of the final polymer product. In direct emulsion polymerization, organic droplets containing reactive monomers or polymers are dispersed in an aqueous solution (oil‐in‐water). Conversely, inverse emulsion polymerization involves dispersing aqueous droplets in an organic medium (water‐in‐oil). Typically, the process has three stages including nucleation, growth of precursor nanoparticles, and polymerization.^[^ ^92^ ^]^ There are two primary methods for this process. In the first method, all reagents are dissolved in the dispersed phase, with photo‐initiators used to start the reaction through homolytic degradation. The second method involves dissolving different monomers in the dispersed and continuous phases. In this approach, droplets usually contain a catalyst and a crosslinker, while the continuous phase includes an initiator. Thermal initiators and reactive species that can be degraded by water radiolysis are commonly used in this setup. The size of the NGs synthesized by this method is influenced by the amount of monomer, pH, and surfactant.^[^ ^77^ ^]^ For instance, Peres et al. synthesized poly(l‐AGA) and poly(l‐AGA*‐co*‐BIS) gel from N,N’‐methylenebis(acrylamide) (BIS) and N‐acryloyl‐l‐glutamic acid (l‐AGA) via inverse emulsion polymerization in which the swelling of the hydrogel was pH dependent;^[^ ^93^ ^]^ 2) RAFT polymerization: It mainly involves dithioester based compounds, which undergoes a series of reaction like chain transfer and reversible addition, etc. without utilizing any metal‐based catalyst.^[^ ^94^ ^]^ The rapid and reversible transfer of chain transfer agents having thio‐carbonyl‐thio groups regulate radical propagation and the development of dormant species during polymerization. This approach significantly extends the lifetime of growing polymer chains while minimizing or eliminating undesirable background polymerization. This is a more controlled technique for NG synthesis among other radical polymerization techniques;^[^ ^95^ ^]^ 3) Click chemistry: For the last few decades, click chemistry‐based crosslinking polymerization technique is used for NG synthesis because this method offers high reactivity, excellent selectivity and high yield.^[^ ^96^ ^]^ In this approach, generally azide and alkyne groups present in the polymer form stable triazole based NG. The reference reactions are those involving copper‐catalyzed and copper‐free strain‐promoted azide‐alkyne cycloadditions.^[^ ^97^ ^]^ According to sharpless, steric effects generally do not influence the reaction, allowing substituted primary, secondary, tertiary, and aromatic azides to readily engage with alkyne‐derived components in the transformation.^[^ ^98^ ^]^ Although, the possible limitation of this approach is the absence of required reactive groups present in the starting materials. However, azides and triple bonds can be readily introduced into polymeric backbones via nucleophilic substitution, yielding highly stable compounds. For instance, crosslinked prodrug NGs were synthesized utilizing polyethylene glycol (PEG)‐modified polypropargyl glutamate and doxorubicin‐functionalized azide via click chemistry reaction to form a three‐dimensional nanoscaffolds for intracellular drug delivery.^[^ ^99^ ^]^ Thiol click chemistry is another “click chemistry”‐based approach for NGs synthesis, which involves thiol‐alkene, thiol‐alkyne, disulfide exchange, and Michael addition reaction. For example, a pH responsive NG was prepared via polymerization from pentaerythritol tetra(3‐mercaptopropionate) methoxy polyethyleneglycol acrylate, and ortho ester diacrylamide for intracellular drug delivery;^[^ ^100^ ^]^ 4) Photo‐induced crosslinking polymerization: It is another popular technique for the synthesis of NG as it induces multifunctionality without adding any crosslinking agent or catalyst which removes the possibility of any by‐product formation.^[^ ^101^ ^]^ Photo‐activatable or dimerizable group functionalized polymers are utilized to create stable NGs architectures through covalent bond formation. This process involves a photo‐initiator, which generates reactive radical species upon exposure to light or through photolysis. The rate at which these initial radicals are produced influences the spatial distribution of covalent crosslinking, thereby affecting the microscopic and macroscopic properties of the resulting nanonetwork, including its swelling behavior, stability, and surface area. Key factors that impact the photo‐crosslinking reaction include the polymer concentration, light intensity, photo‐initiator type and concentration, quantum yield, and the number of radicals generated per photolysis event.^[^ ^102^ ^]^ Although this crosslinking method is facile and efficient, cytotoxicity can be caused by the photo‐initiator.^[^ ^103^ ^]^ For example, a polyvinyl alcohol‐based NGs was synthesized in aqueous medium using Irgacure photo‐initiator, which is well‐tolerated by various cell types.^[^ ^104^ ^]^

The aforementioned crosslinking methods for NG synthesis can also be adapted for flow chemistry synthesis, also known as continuous processing.^[^ ^105^ ^]^ In this method, different reagent streams are pumped into a reactor where a reaction occurs, and the resulting product is collected at the outlet. This approach offers advantages over traditional batch methods, including enhanced heat and mass transfer, improved safety, and greater scalability. Challenges such as solvent compatibility and byproduct formation require inline analysis and purification, while optimizing mass and heat transfer are essential for achieving high efficiency and safety in chemical reactions. For instance, Montalbo synthesized polymeric NGs in a single step using RAFT polymerization within a fluorocarbon microfluidic chip, allowing precise tuning of the NGs sizes by adjusting the flow rate.^[^ ^106^ ^]^

Both physical and chemical crosslinking methods are used to synthesize a wide variety of NGs, but chemical crosslinking is more commonly employed for radiolabeling purpose as the chemical crosslinking method results in a thermodynamically more stable product, which demonstrates better in vivo stability.

## 3 Radiolabeled NGs

**Figure 3:**
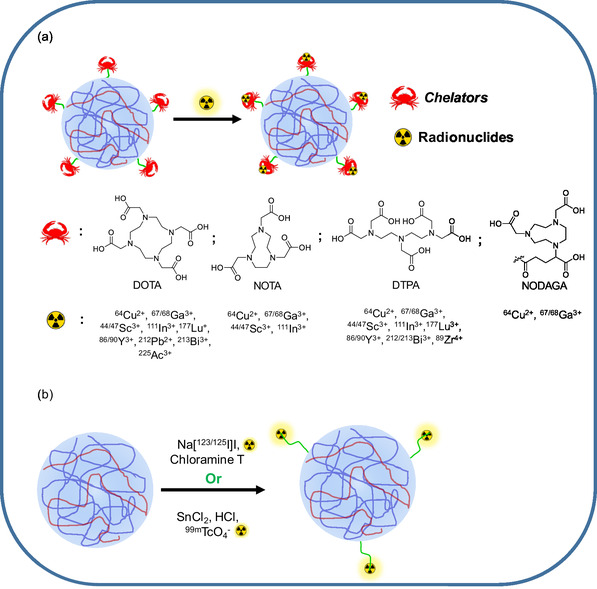
Different strategies used for radiolabeling of NGs. a) Chelator‐based radiolabeling. b) Chelator‐free radiolabeling.

Fundamentally, radiolabeling entails incorporating a radionuclide into the target molecule.^[^ ^107^, ^108^, ^109^, ^110^, ^111^, ^112^ ^]^ Through this method, scientists and professionals can monitor the whereabouts, actions, and interactions of the identified molecule in a system—be it a material, a chemical reaction, or a living entity. Radiolabeling holds particular significance in targeted therapy and diagnostic imaging within nuclear medicine. By attaching a radionuclide to specific molecules like antibodies or nanoparticles, medical professionals can visualize the distribution of these molecules within the body via SPECT or PET imaging techniques.^[^ ^113^, ^114^, ^115^, ^116^ ^]^ This capability facilitates the identification of diseases and the monitoring of treatment effectiveness.^[^ ^117^, ^118^, ^119^, ^120^ ^]^ Also, by labeling the molecules with suitable therapeutic radionuclides, the formulations can be used for targeted therapy.^[^ ^121^, ^122^, ^123^, ^124^ ^]^ In recent years, NGs have been labeled with various radionuclides and utilized for diagnosis and therapeutic purposes.^[^ ^73^, ^125^, ^126^ ^]^ In this section, we shall briefly discuss various radiolabeling strategies for NGs (**Figure** **3**) and potential radionuclides used for the same (**Table** **2**).

**Table 2:** Characteristics and decay modes of various diagnostic (SPECT and PET) radionuclides.^[^ ^137^ ^]^

	Radionuclide	Half‐life	Mode of decay	Principal γ‐component E in keV [% abundance]
SPECT	^67^Ga	3.3 d	EC	93.3 (38.3)
	^99m^Tc	6.0 h	IT	140.5 (88.9)
	^111^In	2.8 d	EC	245.4 (94.2)
	^123^I	13.3 h	EC	159.0 (82.8)
	^201^Tl	72.9 h	EC	167.4 (10.0)
PET	^18^F	109.8 min	β^+^	511 (200.0)
	^44^Sc	3.97 h	β^+^	511 (188.7)
	^45^Ti	3.08 h	β^+^, EC	511 (170.2)
	^52^Mn	5.59 d	β^+^, EC	1434.0 (100.0)
	^64^Cu	12.7 h	β^+^, β^−^, EC	511 (35.6)
	^68^Ga	68.3 min	β^+^, EC	511 (176.0)
	^86^Y	14.7 h	β^+^	1076.6 (82.5)
	^89^Zr	78.4 h	β^+^, EC	908.9 (100)

EC: electron capture, IT: isomeric transition, E: energy.

### 3.1 Radiolabeling Strategies

The polymeric crosslinked structures of NGs allow incorporation of radiometals via two different strategies: 1) chelator‐based radiolabeling; and 2) chelator‐free radiolabeling. Nonmetallic radionuclides like ^18^F and ^123/124/125/131^I are chemically bonded to organic compounds through covalent bonds.^[^ ^127^, ^128^ ^]^ These radiolabeled organic molecules often closely resemble or are identical to their non‐radiolabeled counterparts, behaving pharmacologically in a similar manner. For instance, iodine radioisotopes are commonly used for radiohalogenation processes, with mediators such as chloramine‐T, iodogen, or iodobeads frequently employed for the radioiodination of organic compounds containing tyrosine residues.^[^ ^128^ ^]^ Radioiodination provides advantageous yields, requires fewer synthesis and purification steps compared to metal‐based approaches, and exhibits rapid radiolabeling kinetics. However, these radiolabeling methods sometimes exhibit poor radiochemical stability due to the detachment of radioiodine from the material, which causes undesirable accumulation of the radionuclide in the thyroid glands.^[^ ^129^, ^130^ ^]^ On the contrary, metal‐based radionuclides such as ^99m^Tc, ^67/68^Ga, ^64/67^Cu, ^89^Zr, ^111^In, and ^177^Lu typically necessitate chelators to bind the radionuclide.^[^ ^18^, ^131^ ^]^ Achieving high thermodynamic stability and kinetic inertness is crucial for metal‐based radiotracers to prevent the loss of radiometal in vivo and to avert off‐target accumulation in healthy tissues or organs. Therefore, selecting the optimal chelator for a specific metal ion is of paramount importance, considering various coordination aspects such as coordination number, ionic radii, oxidation state, thermodynamic properties, and electronic properties.^[^ ^18^, ^132^ ^]^ However, the process of chemically modifying NG with a suitable chelator is intricate, requiring prior surface functionalization of NG with functional groups to ensure effective binding with the chelator. This complexity can hinder the radiolabeling process of NGs and extend the time required from initial preparation to the final production of nanoradiopharmaceuticals. In response to these challenges, chelator‐free (intrinsic) radiolabeling methods are now emerging. These approaches not only facilitate rapid, straightforward, and cost‐effective radiolabeling but also maintain the physicochemical properties of NGs to a significant extent.^[^ ^132^, ^133^ ^]^ For instance, Fach et al. synthesized ^103^Pd‐nanogel NG using [^103^Pd][PdCl_4_]^2−^ precursor without the use of chelator.^[^ ^134^ ^]^

### 3.2 Radionuclides

The radionuclides employed in nanoradiopharmaceuticals are categorized based on their application: 1) those utilized for diagnosis or imaging; and 2) those used for targeted therapy. The selection of a radionuclide relies on several factors, including its nuclear decay properties, physical half‐life, ease of production, emission type and energy, emission path‐length, and the type of tumor being treated. Ideally, the physical half‐life of the radionuclide should align with the biological half‐life of the nanoplatform. Moreover, it is crucial for the radionuclide to be clinically producible on a large scale using a cost‐effective and straightforward method that can be routinely implemented. The specific activity of the radionuclide is also vital. Higher specific activity ensures that a lower mass of the nanoplatform is injected, reducing off‐target binding, minimizing receptor saturation, and improving overall targeting efficiency and image quality. Diagnostic radionuclides are selected according to the imaging technique employed, whether it be SPECT or PET. Table 2 summarizes the SPECT and PET radionuclides, which can be utilized for radiolabeling of NGs.

Similarly, depending on the type of emission, the radionuclides are chosen for targeted therapy. **Table** **3** and **4** summarize the commonly used therapeutic radionuclides used in nuclear medicine based on their decay characteristics. Auger electron‐emitting radionuclides have higher linear energy transfer (LET) and a shorter path length (<1 μm). This necessitates the internalization of nanoradiopharmaceuticals (based on the use of Auger electron‐emitting radionuclides) into the nucleus and leads to double strand breaks in DNA, either directly or indirectly via free radical‐mediated pathways.^[^ ^135^ ^]^ Conversely, α‐emitting radionuclides exhibit a tissue range of 50–100 μm with significantly higher LET. Therefore, nanoplatforms labeled with α‐emitting radionuclides do not require internalization and are considered highly efficient in initiating cell apoptosis.^[^ ^136^ ^]^ On the contrary, β^−^‐emitting radionuclides have a lower LET and a longer tissue penetrating range (up to 12 mm). Nanoplatforms labeled with β^−^‐emitting radionuclides similarly do not necessitate cellular internalization for tumor damage.^[^ ^137^ ^]^ However, owing to their extended range, nanoplatforms labeled with β‐emitting radionuclides lead to cell death in surrounding cells via the cross‐fire effect.^[^ ^137^ ^]^

**Table 3:** Nuclear decay characteristics of Auger electron emitting radionuclides.^[^ ^137^ ^]^

Auger electrons (AEs)						Internal conversion (IC) electrons		
Radionuclide	Half‐life	Mode of decay	AEs per decay	Average AE energy/decay [keV]	Average energy per AE [keV]	IC electrons per decay	Average IC electron energy per decay [keV]	Average energy per IC electron [keV]
^67^Ga	78.3 h	EC, γ	5.0	6.6	1.3	0.3	29.7	14.1
^99m^Tc	6 h	IT, γ	4.4	0.9	0.2	1.1	15.2	13.8
^111^In	67 h	EC, γ	7.4	6.9	0.9	0.2	27.9	176.1
^119^Sb	38.2 h	EC	23.7	8.9	0.4	0.8	17	20.2
^123^I	13.3 h	EC	13.7	7.2	0.5	0.2	21.0	222.6
^125^I	60 d	EC	23	12	0.5	0.9	7.3	7.7
^161^Tb	6.9 d	β^−^	0.9	5.1	5.7	1.4	36.7	26.2
^191^Pt	2.8 d	EC	14	17.8	1.3	304	57.1	0.2
^193m^Pt	4.3 d	IT, γ	27.4	10.9	0.4	3.0	126.8	42.4
^195m^Pt	4.0 d	IT, X‐Ray	36.6	23.1	0.6	2.8	161.4	58.1
^201^Tl	73 h	EC, X‐Ray, γ	20.9	14.8	0.7	0.9	29.9	32.9

EC: electron capture, IT: isomeric transition, E: energy.

**Table 4:** Physical characteristics of β^−^ and α emitting radionuclides.^[^ ^137^ ^]^

Type	Radionuclide	Half‐life	Mode of decay	Energy [keV]a)	Principal γ‐component E in keV [% abundance]
α emitter	^211^At	7.2 h	α, γ	5982.4	687.0 (0.3)
	^212^ Bi	60.6 min	α, γ	6207.1	727.2 (11.8)
	^213^ Bi	45.6 min	α, γ	5982.0	439.7 (27.3)
	^223^Ra	11.4 d	α, γ	5979.3	269.4 (13.6)
	^225^Ac	10.0 d	α, γ	5935.1	99.7 (3.5)
β^−^ emitter	^32^ P	14.3 d	β^−^	1710.6	Nil
	^47^Sc	3.3 d	β^−^ γ	600.1	159.4 (68.0)
	^67^Cu	61.8 h	β^−^, γ	577.0	184.6 (48.7)
	^77^ As	38.8 h	β^−^, γ	682.9	239.0 (1.6)
	^89^Sr	50.5 d	β^−^	1496.6	Nil
	^90^Y	64.1 h	β^−^	2282.0	Nil
	^142^Pr	19.1 h	β^−^, γ	2162.3	1575.6 (3.7)
	^153^Sm	46.3 h	β^−^ γ	808.4	103.2 (28.3)
	^166^Ho	26.8 h	β^−^ γ	1854.5	80.6 (6.2)
	^169^Er	9.4 d	β^−^ γ	351.2	Nil
	^175^Yb	4.2 d	β^−^, γ	470.0	396.3 (6.5)
	^177^Lu	6.7 d	β^−^ γ	498.2	208.4 (11.0)
	^186^Re	90.6 h	β^−^, γ	1069.5	137.2 (8.6)
	^188^Re	16.9 h	β^−^ γ	2120.4	155.0 (14.9)
	^194^Ir	19.3 h	β^−^, γ	2246.9	328.4 (13.0)
	^198^Au	2.7 d	β^−^ γ	1372.5	411.8 (95.5)
	^199^Au	3.1 d	β^−^, γ	452.6	158.4 (36.9)

a) Energy of only principal decay mode is mentioned.

**Table** **5** summarizes the radionuclides that have been used for radiolabeling of NGs along with the radiolabeling methods.

**Table 5:** Summary of radionuclides that have been used for radiolabeling of NGs.

NG used	Type of NG	Radionuclides	Radiolabeling method	Application	References
N‐isopropylacrylamide (NIPAAM)‐based NG	Chemically crosslinked NG	^99m^Tc	Chelation using 5‐fluorouracil	Drug delivery in brain tissue	[170]
Lipid carrier‐based 5‐fluorouraci NG with gallic acid‐stearylamin conjugate	Chemically crosslinked NG	^99m^Tc	Chelator‐free radiolabeling using SnCl_2_	Management of pre‐cancerous skin lesions	[172]
Chitosan‐bovine serum albumin‐Carbopol 940 NG	Physically crosslinked NG	^99m^Tc	Chelator‐free radiolabeling using SnCl_2_	Mupirocin dermal delivery	[176]
Polyethylene oxide–polyacrylic acid–folic acid NG	Chemically crosslinked NG	^99m^Tc	Chelator‐free radiolabeling using SnCl_2_	SPECT imaging	[177]
Poly(ethylene oxide‐statpolypropylene oxide based NG	Chemically crosslinked NG	^68^Ga	Chelator‐based radiolabeling using NODAGA chelator	PET imaging	[178]
Thiol functionalized poly(ethylene glycolbased NG	Chemically crosslinked NG	^68^Ga	Chelator‐based radiolabeling using maleimide‐DOTA chelator	PET/CT imaging	[180]
Six arm star‐shaped poly(ethylene glycol) based NG	Chemically crosslinked NG	^68^Ga	Chelator‐based radiolabeling using maleimide‐NODAGA, maleimide‐DOTATATE chelators	Evaluation of tumor penetration efficiency	[182]
Six arm star‐shaped poly(ethylene glycol)‐ based NG	Chemically crosslinked NG	^68^Ga	Chelator‐based radiolabeling using maleimide‐NODAGA chelator	Evaluation of pharmacokinetic profile	[184]
Cholesteryl‐group‐bearing pullulan‐based NG	Physically crosslinked NG	^18^F	Chelator‐free radiolabeling	Protein‐delivery for adjuvant‐free intranasal vaccines	[185]
Cationic cholesteryl‐group–bearing pullulan NG	Physically crosslinked NG	^111^In	Chelator‐based radiolabeling using DOTA chelator	Biodistribution assessment of a nasal vaccine	[186]
Metal‐chelating crosslinkers‐based NG	Chemically crosslinked NG	^64^Cu	Chelator‐based radiolabeling using DTPA, DOTA, and NOTA chelators	PET imaging of tumors and metastases	[187]
Glycol chitosan‐based NG	Chemically crosslinked NG	^64^Cu	Chelator‐based radiolabeling using DOTA chelator	Optical/PET imaging	[193]
Thymidine analog, 5‐iodo‐4”‐thio‐2”‐deoxyuridine	Chemically crosslinked NG	^125^I	Chloroamine T method	Targeted endo‐radio‐therapy	[208]
Six arm star poly(ethylene oxide‐co‐propylene oxide) based NG	Chemically crosslinked NG	^125^I	Chloroamine T method	Delivery of radiopharmaceuticals to brain tumor cells	[126]
Core–shell poly(acrylamide) magnetic NG	Chemically crosslinked NG	^188^Re	Covalent bonding	Targeted radiopharmaceutical applications	[207]
Poly(acrylic acid) based NG	Chemically crosslinked NG	^177^Lu, ^90^Y	Chelator‐based radiolabeling using DOTA chelator	Prostate cancer theranostics	[125]
Poly(acrylic acid) based NG	Chemically crosslinked NG	^177^Lu	Chelator‐based radiolabeling using DOTA chelator	Proof of concept study for potential theranostic applications	[212]
AuPd alloy embedded in gel	Chemically crosslinked NG	^103^Pd	Intrinsic radiolabeling	Nanobrachytherapy	[134]
Polyethyleneimine‐based NG	Chemically crosslinked NG	^131^I	Chelator‐free labeling	SPECT imaging and radiotherapy of breast cancer	[219]
Poly‐N,N’‐dimethyl aminoethyl methacrylate NG	Chemically crosslinked NG	^131^I	Chelator‐free labeling	Radiochemotherapy of cancer	[230]
Carboxymethyl cellulose (CMC) and bovine serum albumin‐based NG	Physically crosslinked NG	^131^I	Chloramine‐T method	Chemo‐radiation therapy of cancer	[231]

## 4 Molecular Imaging Using Radiolabeled NGs

Molecular imaging serves as a noninvasive method in medical diagnosis, providing essential insights into the functional, molecular, and structural processes within an organism.^[^ ^13^, ^138^ ^]^ It enables real‐time visualization of infectious or cancerous diseases with high precision, utilizing both conventional and advanced medical imaging techniques such as optical imaging including fluorescence‐guided surgery and photoacoustic imaging, MRI, CT, and nuclear imaging techniques such as SPECT and PET.^[^ ^13^, ^139^ ^]^ **Table** **6** summarizes the different imaging modalities discussed in this review.

**Table 6:** Summary of different imaging modalities discussed in this review.^[^ ^255^, ^256^ ^]^

Imaging techniques	Source	Spatial resolution	Sensitivity	Depth	Imaging probe	Ionizing radiation effect	Medical information
Optical imaging	Light	Few mm	nM‐pM	mm‐cm	Quantum dots, upconversion nanoparticles, fluorescent dye etc.	No	Structural and functional information
CT	X‐ray	≈100 μm	mM	No limit	High Z elements‐based nanoparticles (Yb, Au etc.)	Yes	Anatomical
MRI	Radio frequency wave	≈100 μm	μM‐mM	No limit	Magnetic nanomaterials	No	Anatomical
SPECT/PET	Gamma rays	Few mm	<pM	No limit	Radionuclides like ^177^Lu, ^198^Au, ^99m^Tc, ^64^Cu, ^68^Ga, ^18^F etc.	Yes	Metabolic

Optical imaging involves detecting light emissions from molecules that have been excited by an external source. These emissions are captured by external cameras and transformed into images. In in vivo applications, optical fluorescence imaging is commonly utilized, leveraging external chemical compounds that emit fluorescence when excited by a specific wavelength of light. This technique allows for imaging of nanoparticles with fluorescent properties, such as quantum dots, upconversion nanoparticles across various spatial scales, from whole‐body imaging to cellular microscopy.^[^ ^140^, ^141^, ^142^ ^]^ These properties also enable this modality for image‐guided drug delivery and image‐guided surgery.^[^ ^143^, ^144^, ^145^, ^146^ ^]^ Depending on the emission wavelength, optical imaging is carried out utilizing three different types of lights: 1) near infrared (NIR); 2) visible; and 3) ultraviolet. Since the last decade, various fluorescent dyes, quantum dots, and silver/gold nanoparticles are being used for optical imaging.^[^ ^147^, ^148^, ^149^, ^150^, ^151^ ^]^ Utilizing NGs for delivering these fluorescent probes improve stability, solubility, and in vivo pharmacokinetics. However, optical imaging has limitations due to the restricted penetration depth of both excitation and emission light through tissues, along with significant tissue autofluorescence, which restrict its use primarily to intraoperative and preclinical settings.

MRI technique utilizes the spin and magnetic properties of certain atomic nuclei, primarily protons (^1^H) found abundantly in water molecules within the body, to generate images.^[^ ^152^ ^]^ The contrast in MRI images is created based on the varying relaxation times of protons in different tissue types, such as fat or blood, which differ in their longitudinal (T_1_) and transverse (T_2_) relaxation times. Nanoparticles containing paramagnetic metals like gadolinium (Gd^3+^) or manganese (Mn^2+/3+^) can alter these relaxation times, enhancing image contrast.^[^ ^153^, ^154^ ^]^ For instance, gadolinium‐based nanoparticles can produce positive T_1_‐weighted contrast, whereas superparamagnetic iron oxide nanoparticles (SPIONs) mainly provide negative T_2_‐weighted contrast but can also offer T_1_‐based contrast depending on their properties.^[^ ^155^, ^156^ ^]^ Additionally, MRI can image other nuclei such as fluorine‐19 (^19^F) when fluorine‐containing nanoparticles are used. Recently, hyperpolarized ^129^Xe biosensors are being used in MRI settings.^[^ ^157^ ^]^ MRI is known for its excellent spatial resolution and its advantage of not using ionizing radiation. Nonetheless, its use in molecular imaging is limited due to relatively low sensitivity and challenges associated with whole‐body imaging and quantitative analysis. Additionally, the contrast agent used in clinical practice may impart harmful side effects, such as non‐biodegradability and toxicity concerns. Considering these, NGs serve as good nanocarriers for contrast agents due to their biocompatibility, high water content and their stability in aqueous medium.^[^ ^158^ ^]^ Moreover, NGs increase the relaxation time and residence of the contrast agent in the blood stream due to decrease in rotational motion and increase in size after contrast agent—polymer conjugation.^[^ ^159^ ^]^ CT scan is another popular non‐invasive molecular imaging technique which utilizes X‐ray irradiation to construct an image depending on X‐ray attenuation signal. While both CT and MRI scans produce 3D images, CT scans offer better spatial resolution, whereas MRI scans provide superior contrast resolution, particularly for soft tissues. As CT contrast agents, iodine molecules, gold nanoparticles, various formulations based on lanthanides, platinum, bismuth, etc. are used.^[^ ^160^, ^161^, ^162^, ^163^ ^]^

Nuclear medicine relies on two primary imaging techniques namely SPECT and PET which enable visualization of physiological changes and aid in diagnosis by detecting radionuclides inside the body.^[^ ^164^ ^]^ In SPECT imaging technique, single gamma ray emitting radionuclides are preferably used to construct 3D images of organs. In most SPECT imaging systems, one to three NaI(Tl) detectors are mounted on gantry and a computer is used for acquisition and processing of data to display the image. PET is another widely used non‐invasive nuclear imaging technique, which require a positron emitting radionuclide combined with a biological molecule. The annihilation of positron with electron leads to emission of two 511 keV γ‐rays to opposite direction, which is detected by the multiple detectors arranged in coincidence mode. The event is registered followed by image reconstruction, which aids in diagnosis. PET is more advantageous compared to SPECT because of its higher spatial resolution. Additionally, a minuscule amount of the probe is required to give an estimation of the biodistribution of the NG in vivo. Over the last few years, the idea of using multiple imaging modalities in conjunction has gained interest and it has been realized that the complementary benefits of different molecular imaging modalities could be harnessed to great effect by using them in‐tandem.^[^ ^165^, ^166^, ^167^ ^]^ For instance, hybrid systems like SPECT/CT, SPECT/MRI, PET/CT, or PET/MRI offer improved specificity with the ability to assess neurologic, endocrinologic, orthopedic, oncologic, cardiac, and infectious diseases. Most of these hybrid imaging modalities are now available in nearly every clinical practice.^[^ ^120^, ^168^, ^169^ ^]^ In the following sections, radiolabeled NGs used for single‐modality (SPECT or PET) imaging and multimodality imaging are discussed in detail and also summarized in Table 5.

### 4.1 SPECT Imaging

The first article on radiolabeled NG for SPECT imaging was published by Soni and co‐workers in 2006.^[^ ^170^ ^]^ N,N'‐methylenebisacrylamide (MBA) NG was synthesized via crosslinking with N‐vinylpyrrolidone (VP) and NIPAM and encapsulated N‐hexylcarbamoyl‐5‐fluorouracil (HCFU), a prodrug of 5‐fluorouracil (FU). The NG was coated with polysorbate 80 for targeted delivery across the blood brain barrier (BBB) and blocking the efflux channel present in tumor cells. HCFU was labeled with ^99m^Tc, followed by encapsulation in an NG matrix.^[^ ^171^ ^]^ Accumulation of radiolabeled NGs in different organs including brain were evaluated in mice and rabbits by accessing the biodistribution pattern and SPECT imaging. High uptake in the reticuloendothelial system (RES) was observed for both polysorbate 80 coated and uncoated radiolabeled NGs, which implied that the stability of ^99m^Tc‐HCFU complex was low under in vivo conditions. However, 1% w/w coating of polysorbate 80 increased the blood circulation time of ^99m^Tc‐labeled HCFU NG and thus enhanced the transportation of NG across BBB compared to the uncoated one. After 5 min of administration of the NG, 0.52%ID of coated NG was accumulated in brain and on the contrary, only 0.18%ID accumulation occurred for the uncoated one. The authors concluded that the polysorbate coating modified the surface property of the NG, which eventually led to higher uptake in the brain.

Rajagopalan et al. also evaluated the biodistribution pattern and pharmacokinetics of ^99m^Tc labeled NG to deliver FU for the treatment of precancerous skin diseases.^[^ ^172^ ^]^ The NG was synthesized by conjugating gallic acid (GA) with stearylamine (SA), leveraging the anticancer properties of GA and the ability of SA to reduce permeability across lipid bilayers. As lipids are already present in the skin, they can induce considerable quantities of drug delivery across the skin barrier as well as enhance its retention.^[^ ^173^, ^174^ ^]^ The authors revealed that ^99m^Tc‐5‐FU NG conjugated with GA‐SA showed higher cytotoxic effect in cancer epidermoid carcinoma cell line (A431) whereas no toxic effect was observed in normal human epidermal keratinocytes (HaCaT) cells.^[^ ^175^ ^]^ The biodistribution pattern after tropical administration of ^99m^Tc‐5‐FU NG in Balb/c mice revealed that the entrapment efficiency was 82.1 ± 1.2%, which was higher than the 5‐FU formulation available commercially.^[^ ^172^ ^]^ Skin to blood localization ratio for ^99m^Tc‐5‐FU NG was 274.93 at 1 h p.i., while that for the commercial formulation was 167.89 at the same time point. The authors suggested that GA‐SA conjugated and 5‐FU loaded NGs possess potential for treating precancerous skin lesions treatment.

In another study, chitosan‐bovine serum albumin based self‐assembled NG was developed to deliver an antibacterial drug mupirocin (MPR) and evaluate its cell binding capability via radiolabeling with ^99m^Tc.^[^ ^176^ ^]^ To enhance the gelatinous property and the adhesive nature of the NG, carbopol 940 was also added to the formulation. The group revealed that >95% of MPR could be entrapped in the NG and almost 93.89 ± 3.07% of the drug was slowly released within 24 h. The NG was also biocompatible in nature and cell binding capacity was also found to be high with or without MPR binding. Moreover, the NG showed its effectiveness against *Staphylococcus aureus*, which made it a potential treatment modality for bacteria induced skin diseases. However, the group did not report any in vivo images.

Utilizing γ‐radiation induced polymerization, a polyethylene oxide and poly‐acrylic acid (PEO–PAA)‐based NG was synthesized by Soliman et al.^[^ ^177^ ^]^ Further, it was radiolabeled with ^99m^Tc without any chelators and followed by electrostatic conjugation with folic acid to form [^99m^Tc]Tc‐PEO‐PAA‐folic acid NG. Biodistribution profile showed high uptake of the radiolabeled NG in ehrlich ascites tumor compared to the normal one, which suggested that [^99m^Tc]Tc‐PEO‐PAA‐folic acid NG is a promising platform for cancer diagnosis.

### 4.2 Positron Emission Tomography (PET)

**Figure 4:**
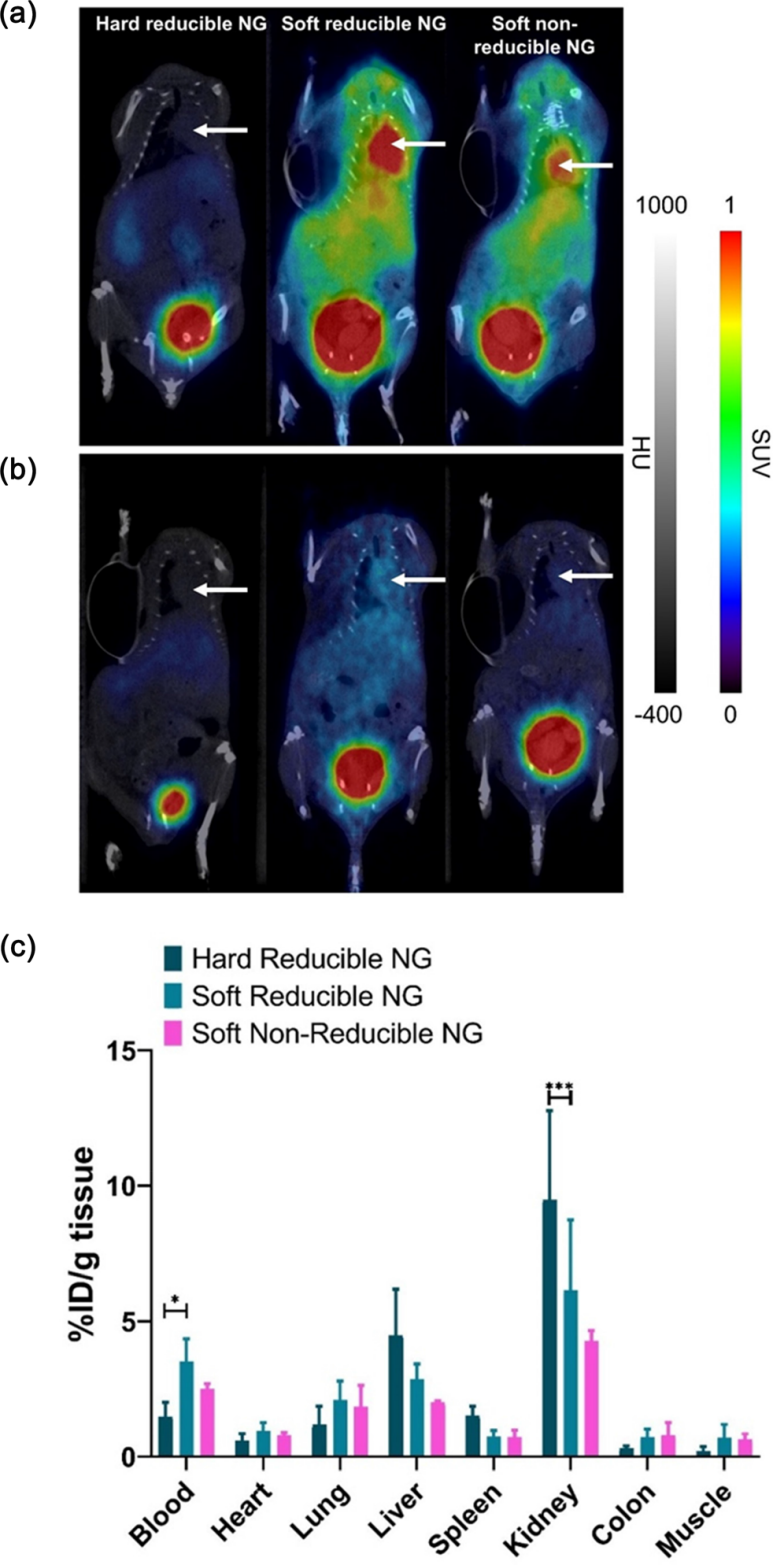
PET/CT images Balb/c nude mice after different radiolabeled NG injections at a) 1 h b) 4 h p.i. SUV; Standard uptake value, HU; Hounsfield units. The white arrows indicate the heart. (c) Biodistribution of different organs after the last PET/CT (4 h p.i.). Reproduced with permission.^[^ ^180^ ^]^ Copyright 2022, Wiley.

Similar to SPECT radionuclides, PET radionuclides were also labeled with NG to track drug delivery and biodistribution in biological systems. Singh et al. synthesized a biodegradable ^68^Ga‐labeled NG in an aqueous medium in the absence of any surfactant or organic solvent.^[^ ^178^ ^]^ The NG was synthesized from star‐shaped poly(ethylene oxide‐statpolypropylene oxide) pre‐polymers and was functionalized with thiol groups. These thiol groups were utilized to covalently link with NODAGA chelator for labeling with ^68^Ga under mild conditions without modifying the NG structure. The NG was internalized by phagocytosis macrophages and was almost unrecognized by the monocytes. Moreover, with additional modification by attaching a targeted moiety, the radiolabeled NG was able to provide an understanding of the in vivo behavior and EPR effect. The major barrier of any nanocarrier including NG is to be opsonized by the mononuclear phagocytic system (MPS).^[^ ^179^ ^]^ Not only the physicochemical properties of the NG influence the circulation time but the mechanical properties also play a major role in this regard.^[^ ^179^ ^]^ Desai et al. demonstrated how tuning the mechanical properties of NG minimizes the phagocytosis process and increase circulation time in peptide receptor radionuclide therapy (PRRT).^[^ ^180^ ^]^ For this purpose, two star‐shaped hard (93 kPa) and soft (37 kPa) reducible NGs were synthesized from thiol functionalized poly ethylene glycol (sPEG‐SH) pre‐polymer. The elastic property of the NGs was modified by varying the number of thiol groups in the precursor without modifying the concentration. Cysteine molecule, present in blood, reduce di‐sulfide to thiols and thus interfere the interaction of NGs in vivo. So as a control, a soft non‐reducible NG was prepared. The in vitro studies in THP‐1 cell lines revealed that higher uptake was observed in the case of hard reducible NG than soft reducible and non‐reducible NGs because hard nanocarriers were phagocytosed at a faster rate.^[^ ^181^ ^]^ This result was also supported by mesoscopic computer simulations studies. In vivo circulation time of the NG in Balb/c mice was also investigated by PET/CT images and biodistribution after radiolabeling the NG with ^68^Ga using maleimide‐DOTA chelators. The PET/CT images demonstrated higher activity in blood suggesting a longer circulation time in the case of soft reducible NGs (**Figure** **4**a,b). Higher blood uptake and lower spleen and liver uptake were observed from the biodistribution profile after 4 h post injection (p.i.) of soft reducible NGs (Figure 4c).

Moreover, higher bladder activity indicates renal clearance of the NG and time dependent decrease in radioactivity in other organs suggests that the NG did not retain but got cleared slowly. The authors concluded that this study demonstrated how changing the elasticity of the NG could influence the cellular uptake pattern and hence drug delivery.

**Figure 5:**
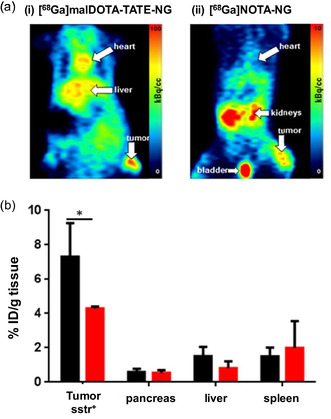
a) PET images at 5 p.i. after intravenous administration of (i) [^68^Ga]malDOTA‐TATE NG and (ii) [^68^Ga]NOTA NG in mice. b) Biodistribution pattern of radioactivity accumulation after administration of [^68^Ga]malDOTA‐TATE NG (presented in black bars) and [^68^Ga]NOTA NG (presented in red bars) in AR42J tumor‐bearing Balb/c mice after 6.5 h p.i. Reproduced with permission.^[^ ^182^ ^]^ Copyright 2017, American Chemical Society.

Drude *et al.* reported how active and passive targeting approaches influence tumor penetration by utilizing radiolabeled NGs.^[^ ^182^ ^]^ A star‐shaped PEG‐based NG was prepared and modified with maleimide‐DOTA‐TATE in a similar way as reported by Singh et al.^[^ ^178^ ^]^ In neuroendocrine tumors somatostatin subtype 2 receptors (SSTR2) are overexpressed and thus somatostatin‐derived peptide (TATE) can induce active targeting.^[^ ^183^ ^]^ The synthesized redox‐sensitive NGs are easily degradable in the tumor microenvironment because of the varying redox potentials compared to glutathione, leading to passive targeting. The NG namely malDOTA‐TATE was radiolabeled with ^68^Ga. In AR42J xenografted Balb/c mice, the pharmacokinetics and the biodistribution pattern of NG were investigated. The uptake in tumor cells, blood circulation, and renal clearance pattern of [^68^Ga]Ga‐malDOTA‐TATE NG was slower compared to [^68^Ga]Ga‐malDOTA‐TATE owing to the larger size of [^68^Ga]Ga‐malDOTA‐TATE NG. Additionally, the NG was degraded to smaller pre‐polymers due to the high intracellular glutathione (GSH) level present in tumor tissues and thus enhanced its penetration depth into the cell layers. A homogeneous distribution of the [^68^Ga]Ga‐malDOTA‐TATE NG was also observed by immunohistological analysis when stained with TATE‐specific antibody. The micro‐PET images demonstrated that higher accumulation of the [^68^Ga]Ga‐malDOTA‐TATE NG was achieved in AR42J xenografted Balb/c mice at 6.5 h p.i. compared to nontargeted NOTA conjugated NG (**Figure** **5**a). The biodistribution pattern at 6.5 h also revealed a similar tumor uptake pattern, that is, 8.2% ± 1.4% and 4.1% ± 1.2%ID/g for [^68^Ga]Ga‐malDOTA‐TATE NG and [^68^Ga]NOTA NG, respectively. (Figure 5b). It was inferred that the combined active and passive targeting strategies using receptor ligand‐modified NG enhanced tumor uptake in homogeneous manner, which might promote future clinical translation.

**Figure 6:**
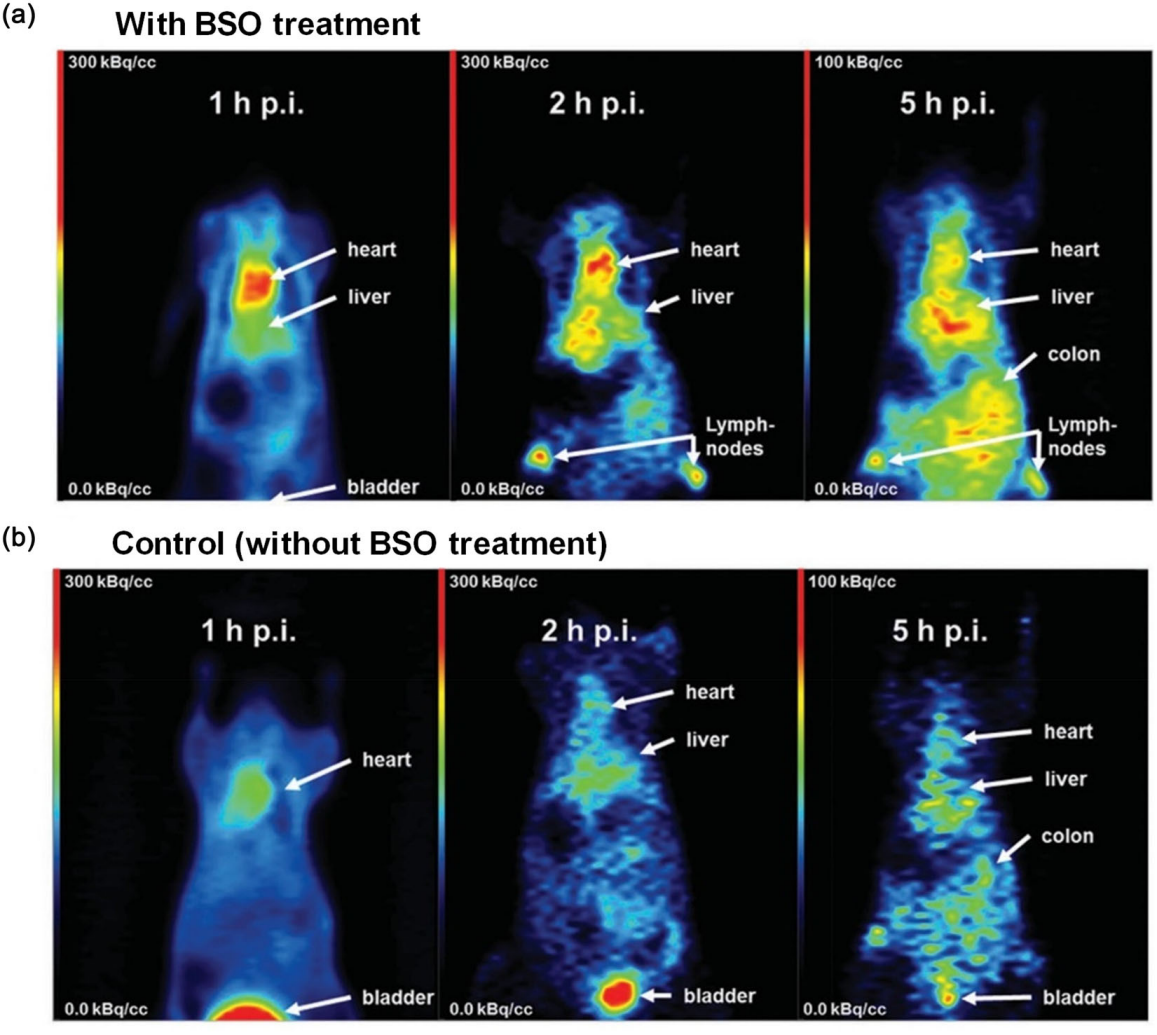
Micro PET images of Balb/c nude mice after intravenous injection of ^68^Ga labeled NG a) BSO and b) without BSO treatment at 1, 2, 5 h p.i. The white arrows indicate heart, liver, kidney, and bladder. Reproduced with permission.^[^ ^184^ ^]^ Copyright 2019, Wiley.

In continuation of the previous study, Drude et al. reported that how GSH causes renal clearance of redox‐sensitive NGs via cleaving disulfide bonds in vivo.^[^ ^184^ ^]^ Interestingly, it was not affected by the size of the NG as a similar clearance pattern was observed for different sizes of NG. Hence, to lower GSH level and inhibit γ‐glutamylcysteine synthetase action, a clinically approved inhibitor buthioninsulfoximin (BSO) was intravenously administered in normal Balb/c mice before administration of ^68^Ga‐NODAGA NG. The PET images (**Figure** **6**) clearly demonstrated how the impact of GSH was influenced by BSO as more than 40% of the elimination of NG was reduced which eventually increased the circulation half‐life of NG. Moreover, pre‐treatment with BSO also enhanced the therapeutic outcome as BSO acted as a radiosensititizer, which increased the level of reactive oxygen species in cancer cells.

A cholesteryl group bearing pullulan (cCHP) and cationic amino group bearing CHP‐based NG was prepared via self‐assembly in an aqueous medium.^[^ ^185^ ^]^ The purpose of this NG was to use as a delivery system for transporting the BoHc/A vaccine subunit of *Clostridium botulinum* type‐A neurotoxin and then release the active portion via an intranasal route. When the drug‐loaded NG was administered, it was not only taken up by the mucosal dendritic cells but also adhered to them because of the strong interaction between cationic NG and the negatively charged cell membrane. To track the performance of the cCHP NG, it was labeled with ^18^F and PET images revealed high accumulation in nasal mucosa. In essence, this NG‐based delivery system created a new route for adjuvant‐free intranasal vaccines.

**Figure 7:**
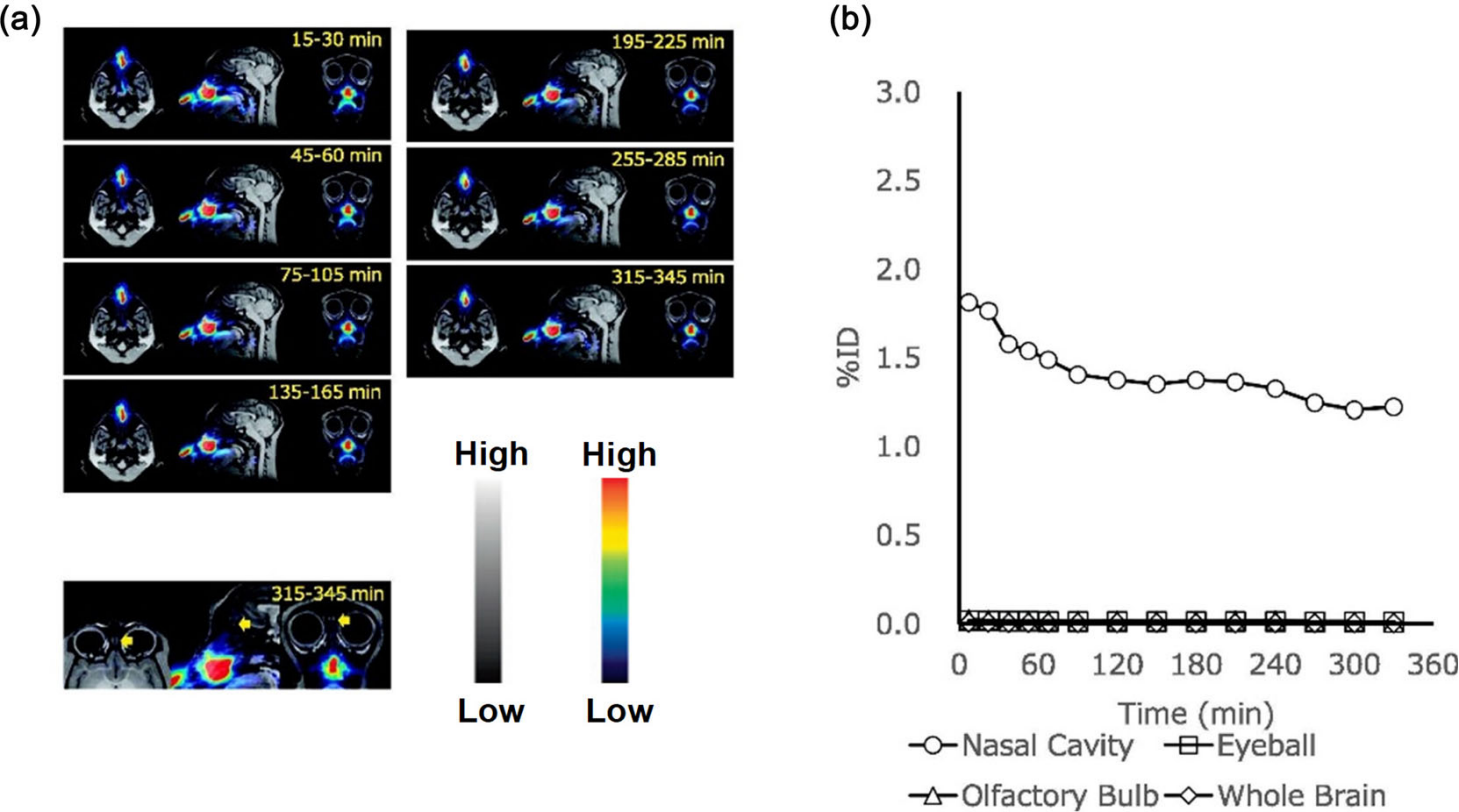
a) PET‐MRI images of a rhesus macaque after nasal administration of [^18^F]cCHP NG. The PET images of the head‐nasopharynx of rhesus macaque are overlaid with MRI scan. The yellow arrows indicate the olfactory bulbs. b) %ID versus time plot for the nasal cavity, olfactory bulbs, and eyes after nasal administration of [^18^F]cCHP NG. Reproduced with permission.^[^ ^186^ ^]^ Copyright 2023, Elsevier.

Another group also synthesized similar cCHP NG for delivering a nasal vaccine (otulinum neurotoxin and pneumococca) and assessed its pharmacokinetics in mice and non‐human primate models.^[^ ^186^ ^]^ This NG might enter the central nervous system via the olfactory bulb due to its close proximity to the nasal cavity. To check the delivery route the NG was radiolabeled with ^111^I (a SPECT radionuclide) and administered via the nasal route in male rhesus macaques and female Balb/c mice respectively. The biodistribution pattern demonstrated predominant uptake of ^111^I was in the nasal passage and feces with a lesser amount in kidney and urine. Moreover, after 72 h of nasal administration, no evidence was found for cerebrum or olfactory bulb uptake. The NG was also labeled with ^18^F to investigate the biodistribution pattern of the vaccine. The PET images of rhesus macaques after administration of [^18^F]cCHP NG ensured that no NG uptake was observed in the cerebrum, olfactory bulbs, or eyes after 6 h of administration (**Figure** **7**). The authors inferred that cCHP NG was a promising delivery system for nasal vaccine supported by both ex vivo biodistribution and PET imaging studies.

**Figure 8:**
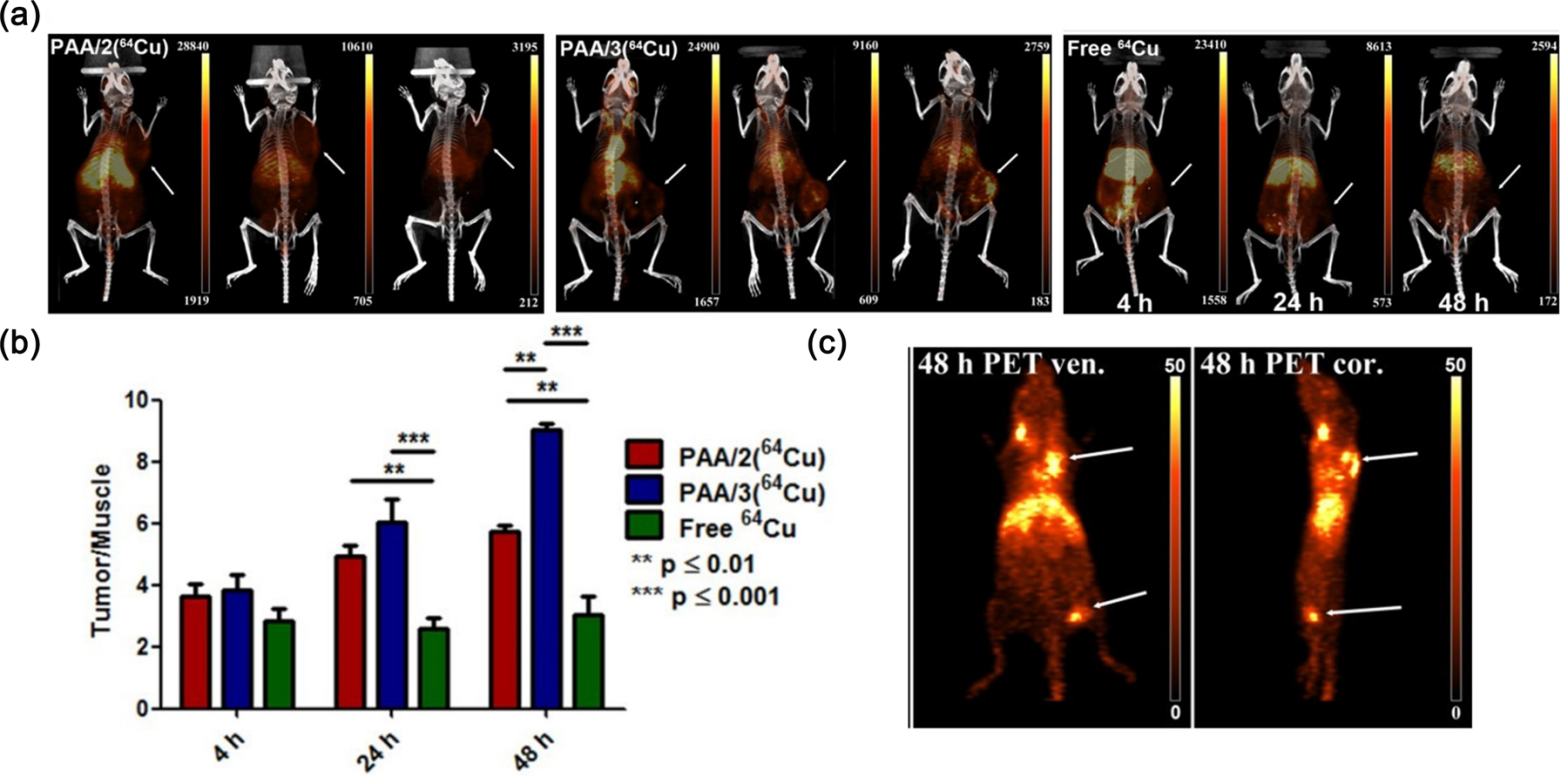
a) PET/CT images of 4T1 tumor‐bearing mice at 4, 24, 48 h p.i. with ^64^Cu‐labeled NG crosslinked with DOTA (PAA/2), NG crosslinked with NOTA (PAA/3) and free ^64^Cu^2+^. Arrows indicate tumor position. b) PET signal of tumor/muscle ratio. c) PET images of tumor on shoulder region and popliteal lymph node in leg at 48 p.i. Reproduced with permission.^[^ ^187^ ^]^ Copyright 2015, Ivyspring International Publisher.

In another report, Lux et al. formulated metal chelating crosslinked polyacrylamide‐based (PAAm) NG for incorporating PET radionuclide ^64^Cu, which could be a potential PET probe.^[^ ^187^ ^]^ Three different chelators namely DTPA, DOTA, and NOTA were individually conjugated with NG via crosslinking and labeled with ^64^Cu. Among them, the highest chelating ability was achieved using NOTA and ≈94% of ^64^Cu was retained in the NG even after 48 h of incubation in serum medium. Stability and uptake of the NG were demonstrated in 4T1 murine mammary tumor‐bearing BALB/c mice by injecting ≈6.6 MBq of ^64^Cu labeled DOTA (PAA/2) and NOTA (PAA/3) crosslinked NG. The highest tumor accumulation was observed for PAA/3(^64^Cu), which increased from 2.9 to 6.95%ID/g at 48 h p.i. (**Figure** **8**a). Moreover, the tumor/muscle intensity ratio was >9 after 48 h of injection (Figure 8b). In addition, the radiolabeled NG was also able to identify metastasis other than the primary tumor signifying its potential for clinical translation (Figure 8c). The authors suggested that this NG platform can be used for bimodal PET/MRI imaging by incorporating both ^64^Cu‐NOTA and earlier reported Gd^3+^‐DOTA crosslinker for improving diagnosis.^[^ ^188^, ^189^ ^]^

### 4.3 Multimodality Imaging

The future of cancer patient care lies in molecular or personalized medicine, and molecular imaging is essential to achieve this objective.^[^ ^13^, ^190^ ^]^ Nevertheless, no single molecular imaging method is ideal or adequate to provide all the information required. For example, it is challenging to quantify optical fluorescence imaging, particularly in tissue that is deeper than a few millimeters within a subject; PET offers very high sensitivity but poor resolution, and MRI has excellent soft tissue contrast but low sensitivity.^[^ ^191^, ^192^ ^]^ However, in MRI and CT imaging, comparatively higher amount of contrast agents are used but in nuclear imaging techniques, a radiotracer is used that minimizes the disturbance to the biological system and reduces the likelihood of inducing toxic effects. Additionally, radionuclides enable precise quantification of nanoparticles tissue uptake both in vivo and ex vivo, with high temporal resolution. This capability is crucial and often difficult to achieve with MRI or CT, making nuclear imaging particularly useful for comprehensive whole‐body analysis of nanoparticles pharmacokinetics and biodistribution. Therefore, combining multiple imaging modalities is clearly attractive when interrogating an individual because a single modality cannot provide information on every element of anatomy and function. By combining these modalities, researchers and clinicians can achieve a more accurate and detailed understanding of complex biological systems and disease mechanisms, enhance diagnostic accuracy, and improve the evaluation of therapeutic interventions. This synergistic approach facilitates a more holistic assessment of health conditions, leading to better‐informed clinical decisions and advancements in medical research. In light of this, multimodality imaging using radiolabeled NGs is discussed in this section.

**Figure 9:**
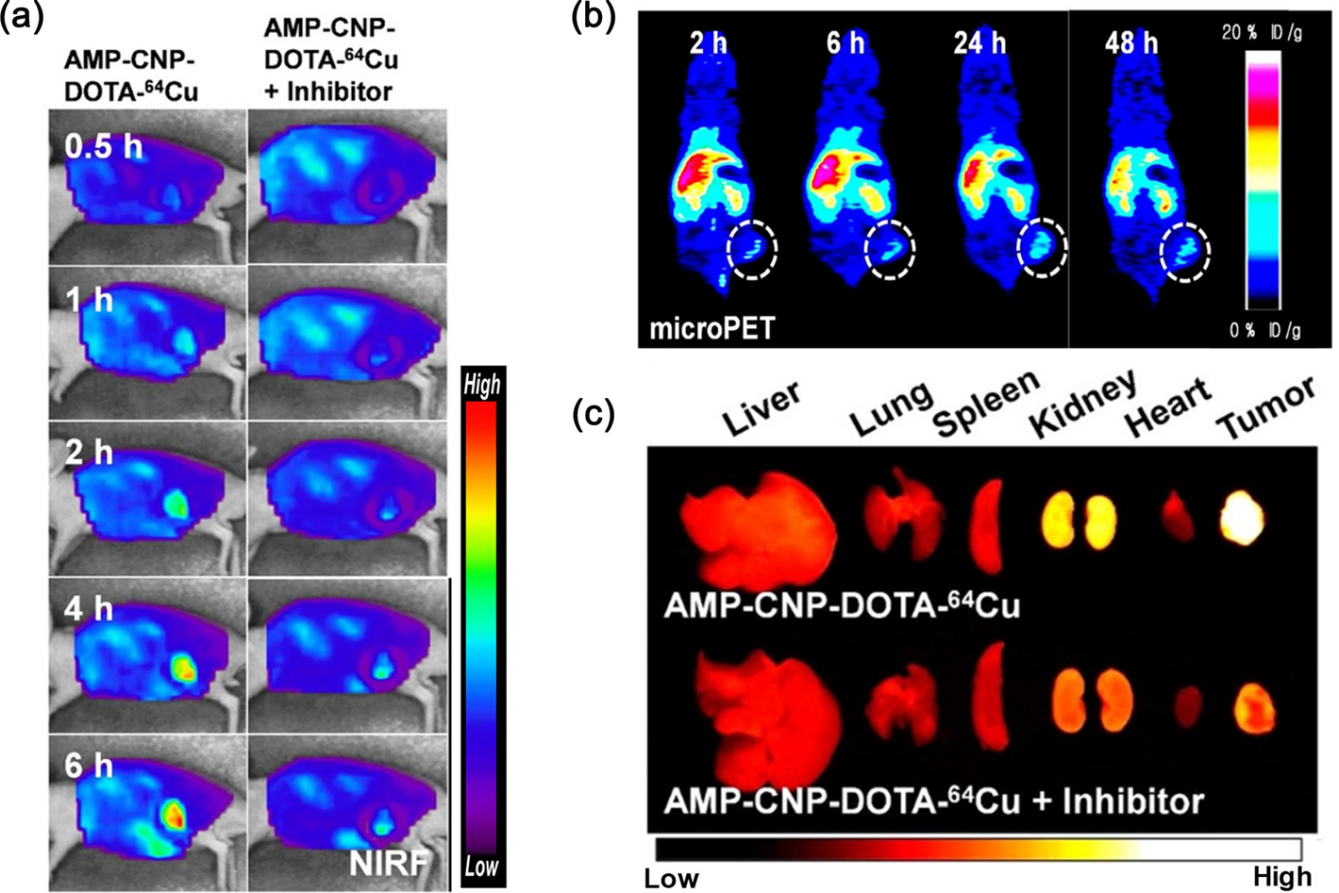
Whole body in vivo a) NIRF and b) micro‐PET images at different time points. White circles indicate the position of tumor. c) Ex vivo fluorescence images of different organs of A549 tumor‐bearing mice at 6 h. Reproduced with permission.^[^ ^193^ ^]^ Copyright 2014, American Chemical Society.

The first dual PET and optical imaging probe was reported by Lee et al. in which a metalloprotease‐activated glycol chitosan nanoparticle (CNP) was formulated.^[^ ^193^ ^]^ For that purpose, glycol chitosan was modified with azide groups and 5β‐cholanic acid, and self‐assembled to nanoparticle via hydrophobic interaction. By employing click chemistry, a dibenzocyclooctyne functionalized DOTA (DOTA‐Lys‐PEG_4_‐DBCO) crosslinker was used to conjugate ^64^Cu and an activated peptide specific metalloproteinase (MMP) matrix was tagged with a fluorophore Cy5.5 (AMP‐DBCO) and a dark quencher molecule (BHQ3). The MMP specific receptors are overexpressed in many tumors and cause tumor progression and metastases.^[^ ^194^, ^195^ ^]^ The radiolabeling of ^64^Cu was performed before DOTA ‐ AMP‐DBCO conjugation via alkyne–azide cycloaddition. To validate the multimodality imaging, AMP‐CNP‐DOTA‐^64^Cu was intravenously injected into A549 tumor‐bearing mice. The near‐infrared fluorescence (NIRF) signal was detected after 1 h p.i. and the signal intensity gradually increased with time (**Figure** **9**a). However, the NIRF signal intensity from the tumor was reduced when the MNP inhibitor was used, as fluorescence quenching only occurred after the peptide was cleaved by MNP. The whole‐body PET images in Figure 9b demonstrated a gradual increase in uptake of [^64^Cu]Cu‐DOTA‐AMP‐CNP in tumor and the uptake saturated at 24 h p.i. (6.2%ID/g.) Although high uptake of [^64^Cu]Cu‐DOTA‐AMP‐CNP was observed in kidney and liver at early time points, it gradually decreased at 48 h p.i. Biodistribution study performed after 48 h of intravenous injection also corroborated the conclusions drawn from dual modality imaging. Nearly 15%ID/g of the nanoparticle was found in liver, 10%ID/g, 15%ID/g, and 9%ID/g uptake were found in spleen, kidney, and blood, respectively. Ex vivo NIRF imaging of the tumor and different organs was carried out to confirm the observation of in vivo dual modality imaging (Figure 9c). Stronger signal intensity was observed in the tumor region than the inhibitor treated tumor, which was in good agreement with the whole‐body imaging. However, the signal intensity in the kidney was much higher in ex vivo imaging than in vivo due to excretion of fragmented fluorescent dye (Cy5.5) dissociated from MNP. Highest signal intensity of MMP and tumor accumulation of radiolabeled nanoparticle was observed by both optical and PET imaging, which could improve the accuracy of tumor diagnosis.

## 5 Targeted Radiotherapy Using Radiolabeled NGs

While conventional cancer therapies such as immunotherapy, hormone therapy, external radiation therapy, chemotherapy, and use of angiogenesis inhibitors have made great progress against different types of cancers, they all have inherent drawbacks that highlight the continuous need for innovation in the field.^[^ ^196^, ^197^, ^198^, ^199^, ^200^ ^]^ Chemotherapy can be quite efficient in stopping the rapid division of cancer cells, but because it is nonspecific, it can also have detrimental effects on healthy cells in addition to cancerous ones.^[^ ^201^ ^]^ Hormone resistance can develop over time as a result of hormone therapy, which is frequently used to treat hormone‐sensitive malignancies like breast and prostate cancer.^[^ ^202^ ^]^ External radiation therapy can cause long‐term consequences by damaging nearby healthy tissue and organs, even while it is effective in reducing tumor size and killing cancer cells.^[^ ^203^, ^204^ ^]^ Immunotherapy exploits the immune system of the body to combat cancer, although it is not always effective and resistance to the therapy might develop depending on the patient's condition and the type of cancer.^[^ ^205^ ^]^ Angiogenesis inhibitors prevent new blood vessels from growing, but with time, tumors may become resistant to them.^[^ ^199^ ^]^ Thus, each therapeutic modality has its own limitations, which emphasize the necessity for ongoing research into more targeted and individualized strategies. Potential drug delivery methods (including delivery of therapeutic radionuclides) using NGs have been suggested as a way to circumvent the shortcomings of current conventional cancer treatments.^[^ ^55^, ^206^ ^]^ NGs have been envisioned as perfect drug delivery systems, possessing great stability and huge drug loading capacity. It is possible to alter NGs to improve drug accumulation at disease locations and accomplish active targeting. Additionally, they can be made to respond to both internal and external stimuli, which reduces the pharmacological side effects and drug build‐up in non‐target tissues.^[^ ^206^ ^]^ A summary of targeted radiotherapy utilizing radiolabeled NGs is also summarized in Table 5.

Sun et al. reported a potential core–shell NG formulation for radiopharmaceutical application in 2004.^[^ ^207^ ^]^ In the formulation, superparamagnetic Fe_3_O_4_ nanoparticles were encapsulated in polyacrylamide (PAM) in emulsion‐free aqueous medium by UV light irradiation where N,N_o_‐Methylenebis‐(acrylamide) acted as a cross linker. The surface of the NG was modified by carbonyl elimination via Hoffmann degradation for protein immobilization. This magnetic NG was labeled with ^188^Re via L‐histidine immobilization and exhibited over 83% retention of radioactivity after 48 h incubation in calf serum. Overall, this magnetic NG was proposed as a promising carrier for therapeutic radionuclides for cancer treatment.

Morgenroth and group investigated the therapeutic efficacy of thymidine analog, 5‐iodo‐4”‐thio‐2”‐deoxyuridine labeled with ^125^I ([^125^I]ITdU) to treat multiple myeloma.^[^ ^208^ ^]^ The Auger electron emitter, ^125^I is highly radiotoxic when incorporated into DNA of cancer cells.^[^ ^209^ ^]^ To achieve DNA proximity, ^125^I should be tagged to nucleoside analogs like thymidine, which act as a vehicle to tumor cells. Due to the presence of blood brain barrier (BBB) and poor selectivity, the effectiveness of the drug is limited. To address this issue, the same group synthesized a stimuli responsive NG‐based carrier system to deliver ([^125^I]ITdU) to treat glioma.^[^ ^126^ ^]^ In this study, poly(ethylene oxide*‐co*‐propylene oxide) pre‐polymers having acrylate groups were used as a building block to synthesize NG which was further conjugated with [^125^I]ITdU‐MMP2/9. MMP‐2 and MMP‐9 could induce enzymatic degradation of NG and subsequently release [^125^I]ITdU in cancer cells. Abundance of MMP‐2 and MMP‐9 is mostly found in HT12346 and U‐87 glioblastoma cell lines.^[^ ^210^ ^]^ To persuade BBB and delivery of NG into cell, the NG was functionalized post synthesis with cross‐reactive material 197 (CRM‐197). CRM‐197 is a clinically approved ligand for addressing diphtheria toxin receptor (DTR) and facilitates efficient delivery across BBB toward the targeted site.^[^ ^211^ ^]^ In vitro studies in human glioblastoma cell lines confirmed transcytosis across the BBB. Efficient release of [^125^I]ITdU and high rate of DNA incorporation were achieved in glioblastoma cell lines. This approach of glioblastoma cell killing might be a new strategy for cancer care. However, the authors did not report any in vivo studies.

In another study, Matusiak et al. developed a poly(acrylic acid) (PAA) based targeted NG system for use as radionuclide carrier.^[^ ^212^ ^]^ PAA NG was synthesized by electron pulses generated from a linear accelerator in acidic condition (pH ≈ 2). Then, it was conjugated with a DOTA bombesin derivative. This bombesin derivative has high affinity toward gastrin‐releasing peptide receptor (GRPR) which is overexpressed in various kind of cancerous tumors such as, breast, pancreas, and prostate.^[^ ^213^ ^]^ Hence, this DOTA – bombesin derivative would not only chelate with various radionuclides but also enhanced target specificity. As a proof of concept, NG was labeled with ^177^Lu and >95% radiolabeling yield was achieved. In continuation of the previous study, the same group reported another PAA based NG system for delivering radionuclides to treat prostate cancer.^[^ ^125^ ^]^ In this report, PAA NG was synthesized in a similar way as described above and then functionalized with Lys1Lys3‐bombesin. After that, it was labeled with ^177^Lu and ^90^Y using DOTA chelators and the radiolabeled NGs demonstrated high stability in human serum and radiolabeling buffer medium up to 14 days. The in vitro study in PC‐3 cell lines revealed nearly 20% of radioactivity was internalized after 4 h of incubation, which was significantly greater than the non‐targeting counterpart. The normal animal biodistribution pattern after intravenous administration of radiolabeled NG revealed 50%ID/g uptake in liver and spleen even after 7 days whereas less than 5% uptake was observed in other organs. Despite the fact that liver is the responsible organ for nanoparticle metabolization, high liver uptake would cause significant radiotoxicity inducing DNA damage and reactive oxygen species generation.^[^ ^214^, ^215^ ^]^ Additionally, only 1%ID/g tumor uptake was achieved after intravenous injection of both radiolabeled NGs in PC‐3 tumor‐bearing mice. Hence, the authors concluded that the studied NG systems required further improvement in terms of tumor uptake, biodistribution and clearance pattern.

**Figure 10:**
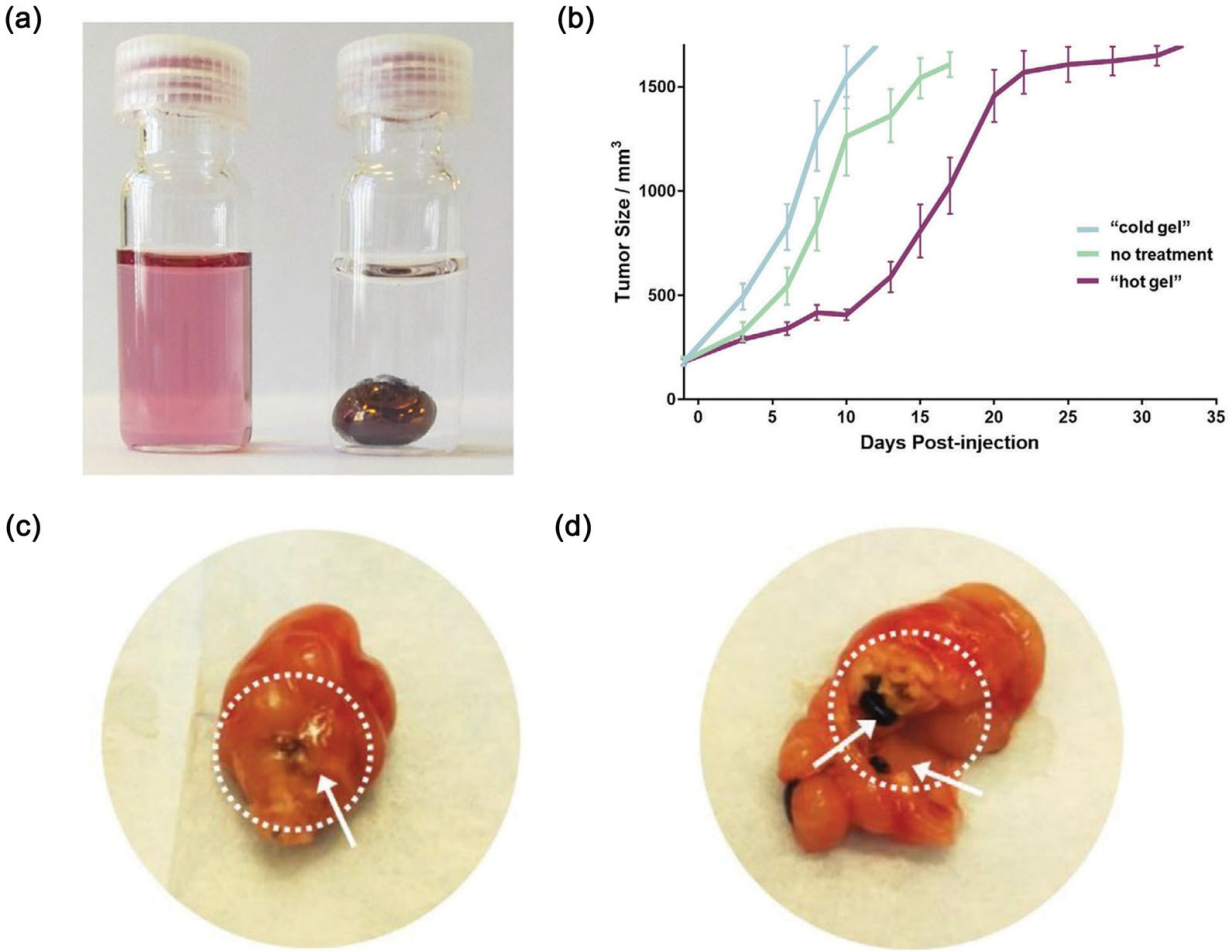
a) Effect of different medium on NG. In ethanol medium the formulation dissolved instantly (left) whereas in PBS medium a stable droplet is formed (right). b) Therapeutic efficacy of CT26 tumor‐bearing mice treated with different NG formulations. c) Image of isolated tumor after intratumoral administration of ^103^Pd‐NG where the visibility of NG is indicated by white arrow. d) The white arrows indicate separated NGs from tumor tissue and no further growth in tumor periphery. Reproduced with permission.^[^ ^134^ ^]^ Copyright 2021, Wiley.

Fach et al. reported an interesting article about [^103^Pd]AuPd nanoparticle embedded in gel matrix for nanoscale brachytherapy.^[^ ^134^ ^]^ A radioactive precursor of [^103^Pd]Pd^2+^ was reduced by sodium citrate in presence of Au^3+^ solution resulting into [^103^Pd]AuPd nanoparticles followed by coated with poly(N‐isopropylacrylamide) to make it dispersible. The resulting formulation was further mixed with sucrose acetate isobutyrate (SAIB) or lactose octaisobutyrate (LOIB) in ethanol medium to make biocompatible and injectable nanoformulation. Both SAIB and LOIB are biocompatible esters and already been used as a scaffold for nanoparticle immobilization, radiotherapy, drug delivery.^[^ ^216^, ^217^, ^218^ ^]^ The radiolabeled NG formulation readily dissolved in ethanol medium whereas in PBS medium a stable insoluble structure was formed (**Figure** **10**a), which displayed retention of 99.97% of radioactivity for 25 days. This NG encapsulated formulation delayed tumor growth within 10 days of intratumoral administration in CT26 colorectal cancer tumor‐bearing mice (Figure 10b). The tissue surrounding the tumor region became soft due to the radiation damage and ^103^Pd‐NG was easily separated from the tumor implying the therapeutic efficacy of the NG (Figure 10c,d). Moreover, 54 ± 13%ID/g tumor uptake of the radiolabeled NG was observed after 20 days of treatment and negligible amount (0.01%ID/g) of the formulation was found in other organs.

**Figure 11:**
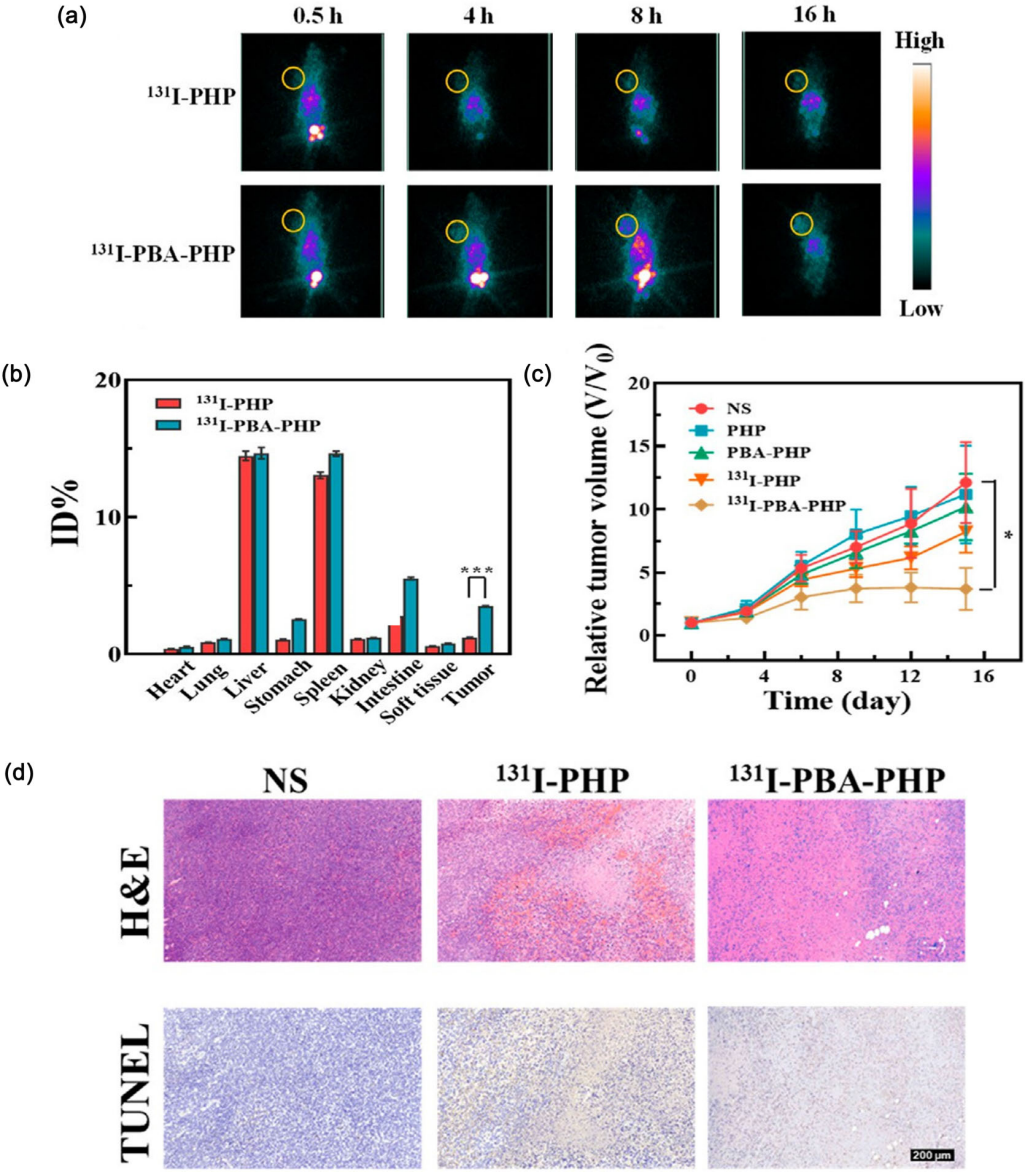
a) SPECT images of tumor‐bearing mice after injection with ^131^I‐PHP NGs and ^131^I‐PBA‐PHP at different time points; b) Change in signal intensities of SPECT of major organs 16 h p.i.; c) Plot of change in relative tumor volume; d) Representative H&E‐stained images of tumor after different treatments (scale bar 200 μm). Reproduced with permission.^[^ ^219^ ^]^ Copyright 2022, Frontiers.

Another NG‐based theranostic nanoplatform was reported by Kong et al.^[^ ^219^ ^]^ The NG was developed from polyethyleneimine (PEI) by crosslinking with 3‐(4′hydroxyphenyl)propionic acid N‐hydroxysuccinimide (HPAO) by water/oil polymerization. To target sialylated epitopes overexpressed on the surface of various tumors like breast cancer, the PEI‐based NG (P.NH_2_ NG) was linked with phenylboronic acid (PBA). Finally, ^131^I was conjugated with the acylated group of PEIs to obtain ^131^I‐PBA‐PHP NG. SPECT images were recorded after intravenous injection of ^131^I‐PHP and ^131^I‐PBA‐PHP NG in 4T1 tumor‐bearing Balb/c nude mice (**Figure** **11**a). The data revealed that the uptake of the ^131^I‐PBA‐PHP NG peaked after 8 h p.i. whereas ^131^I‐PHP NGs treated mice displayed weak signal. Although the result obtained from the SPECT signal intensities (Figure 11b) and biodistribution studies revealed that both PBA conjugated NG accumulated in tumor, heart, lung, stomach, kidney, and soft tissues, higher accumulation in tumor was observed in case of targeted ^131^I‐PBA‐PHP NG. Moreover, slowest tumor growth was observed for the mice treated with targeted radiolabeled NG, that is, ^131^I‐PBA‐PHP NG (Figure 11c). Notably, prolonged survival with negligible change in body weight were observed for the mice treated with radiolabeled PBA‐PHP NG. The large region of tumor apoptosis and necrosis were also apparent from terminal deoxynucleotidyl transferase dUTP nick end labeling (TUNEL) and hematoxylin and eosin (H&E) stained images, as shown in Figure 11d. The authors envisaged that the NG labeled with ^131^I was a promising platform for cancer treatment.

## 6 Combination Therapy Using Radiolabeled NGs

Advancements in nanotechnology have revolutionized cancer treatment by enabling the development of nanocarriers capable of co‐administering multiple therapeutic agents and delivering them precisely to the desired site.^[^ ^220^ ^]^ This combination therapeutic approach offers several significant advantages over single‐modality treatments. Firstly, it allows for the simultaneous delivery of different drugs with complementary mechanisms of action, thereby enhancing the therapeutic efficacy against cancer cells while minimizing toxicity to healthy tissues. By targeting multiple pathways involved in cancer progression or resistance simultaneously, combination therapy can achieve synergistic effects that surpass those of individual drugs alone.^[^ ^221^, ^222^, ^223^, ^224^, ^225^ ^]^ Furthermore, the capacity to administer several therapeutic agents simultaneously reduces the total dosage of each medication required lowering the possibility of adverse side effects and enhancing patient tolerance to the drug. This diverse approach to cancer treatment highlights how promising nanotechnology is for improving treatment outcomes and reducing the drawbacks of conventional cancer medicines.^[^ ^226^, ^227^ ^]^ The efficacy and safety of cancer therapy regimens could be greatly enhanced by further optimizing drug combination methods and nanocarrier design as this field of study progresses. Radiolabeled NGs are excellent platforms for co‐delivery of drugs and therapeutic radionuclides due to their 3D network structure, which are able to incorporate both drugs and radionuclides into their cavity.^[^ ^7^ ^]^

**Figure 12:**
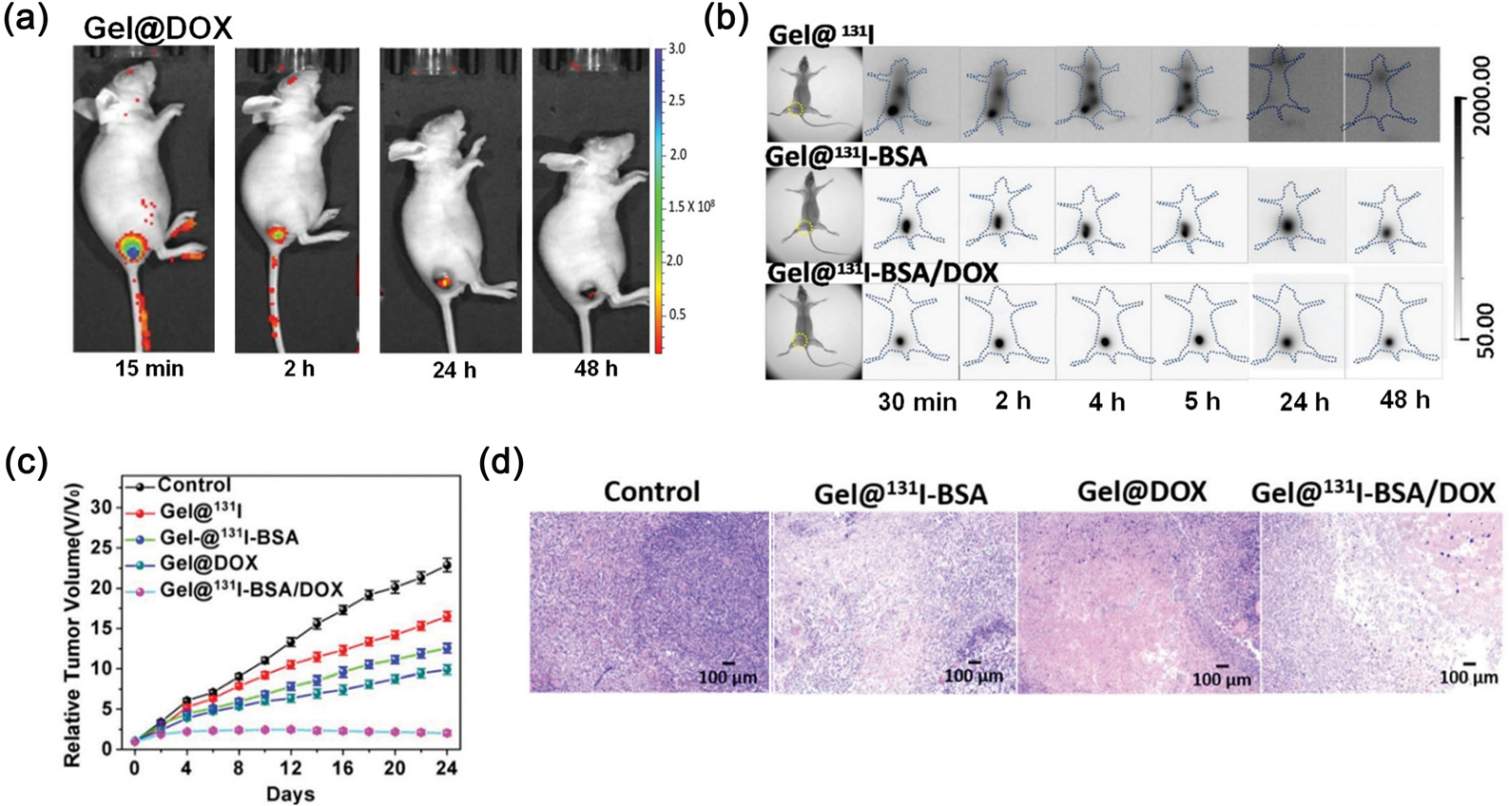
a) Optical imaging of 4T1 tumor‐bearing mice after intratumoral injection of Gel@DOX. b) γ ‐ imaging of 4T1 tumor‐bearing mice after intratumoral injection of Gel@^131^I, Gel@^131^I‐BSA, and Gel@^131^I‐BSA/DOX at different time points. c) Tumor growth plot of 4T1 tumor‐bearing mice after intratumoral injection of various agents. d) Histological analysis of tumor slice 1 day post treatment. Reproduced with permission.^[^ ^230^ ^]^ Copyright 2018, Royal Society of Chemistry.

Chemo‐radiotherapy is one such combination therapy that is widely used by clinicians for cancer treatment.^[^ ^228^, ^229^ ^]^ In this direction, Maiti *et al.* synthesized a thermosensitive, injectable poly‐N,N’‐dimethyl aminoethyl methacrylate (PDMAEMA) NG.^[^ ^230^ ^]^ PDMAEMA was prepared by chemical crosslinking route adopting free radical precipitation polymerization (FRPP) method where DMAEMA (N,N’‐dimethyl aminoethyl methacrylate), ammonium persulfate and N,N′‐methylene‐bis‐acryl amide were used as monomeric unit, initiator and crosslinking agent, respectively. At physiological temperature (37 °C), PDMAEMA was able to form gel via hydrophobic interaction. A chemotherapeutic drug doxorubicin (DOX) and ^131^I (therapeutic radionuclide) labeled bovine serum albumin were loaded in PDMAEMA (PDMAEMA@^131^I‐BSA/DOX) and its efficacy was demonstrated in tumor‐bearing mouse model. Before radiotherapy, in vivo DOX release was evaluated by intratumorally injecting free DOX and Gel@DOX in 4T1 murine breast tumor‐bearing mice. The fluorescence images demonstrated gradual release of DOX from Gel@DOX whereas no free DOX was detected beyond 1 h p.i. (**Figure** **12**a). Gamma imaging after intratumoral injection of Gel@^131^I‐BSA/DOX demonstrated prolonged retention in tumor site suggesting strong interaction between gel and BSA (Figure 12b), which was also supported by biodistribution studies. Dual chemo‐radiotherapy of the NG was demonstrated in mice model by intratumoral injection of Gel@DOX (50 μg mL^−1^ of DOX), Gel@^131^I‐BSA (200 μCi of ^131^I) and Gel@^131^I‐BSA/DOX (200 μCi of ^131^I and 50 μg mL^−1^ of DOX) and observed for 24 days. Although the mice treated with Gel@DOX and Gel@^131^I‐BSA showed reduction in tumor growth rate initially, after 1 week, the tumor growth increased sharply. On the contrary, tumor growth was significantly retarded for the group treated with Gel@^131^I‐BSA/DOX because of gradual release of DOX along with the prolonged retention of ^131^I at the tumor site (Figure 12c). In addition, the body weight of the mice did not change notably suggesting minimal side effect. The histological analysis demonstrated that highest level of necrosis in the tumor was observed for the group treated by combination therapy compared to other treatment groups (Figure 12d). The authors concluded that temperature stimulated gel formation property and pH sensitive drug releasing behavior of the NG governed the retention of the radionuclide and gradual release of drug within the diseased site, respectively. Finally, the collective therapeutic outcome rather than individual chemo or radiotherapy is a promising platform for cancer treatment with lesser side effects compared to conventional chemo or radiotherapy.

Another chemo‐radiotherapeutic NG was prepared for cancer therapy by Liu and co‐workers.^[^ ^231^ ^]^ Hybrid self‐assembled bovine serum albumin (BSA) and carboxymethyl cellulose (CMC) made NG was developed by adopting a simple electrostatic approach for co‐delivery of ^131^I radionuclide and chemotherapeutic drug camptothecin (CPT). The opposite charge of BSA and CMC in acidic pH range played a major role for the formation of NG. Moreover, intermolecular hydrophobic association occurred when the temperature increased more than protein denaturation temperature, which also induced association of CMC and BSA. The drug CPT was encapsulated in NG via hydrophobic interaction with BSA, a well‐known biocompatible hydrophobic material for drug carrier. The NG exhibited ≈89.42% encapsulation efficiency and the drug loading capacity was ≈16.72 wt%. In vitro drug release profile of ^131^I‐BSA‐CMC/CPT exhibited sustained release at both pH 5.0 and 7.4. Interestingly, the drug was first released at pH 5.0 and again at pH 7.4, which is favorable for minimizing damage to normal tissues. Furthermore, the NG also exhibited high biocompatibility up to concentration of 1000 μg mL^−1^, when incubated in Lewis lung carcinoma (LLC) cell lines. Additionally, ^131^I‐BSA‐CMC/CPT circulated in blood for prolonged period of time compared to free ^131^I when administered in normal C57BL6 mice via intravenous injection. High uptake of ^131^I‐BSA‐CMC/CPT was observed in tumor site even after 72 h p.i. whereas fast clearance was observed from other organs. LLC tumor‐bearing mice treated with ^131^I‐BSA‐CMC/CPT NG (300 μCi of ^131^I) inhibited tumor growth significantly which was better than mice treated with BSA/CMC‐CPT and ^131^I‐BSA/CMC. Overall, this biocompatible NG prepared via eco‐friendly method is an excellent platform, which is able to offer a distinct opportunity for chemo‐radiotherapy of cancer.

## 7 Challenges and Possible Strategies for Clinical Translation of Radiolabeled NGs

Since their discovery, NGs have witnessed tremendous development in the biomedical field in applications ranging from drug delivery to non‐invasive imaging and diagnosis due to their superior biocompatibility, biodegradability, and non‐toxic nature over several other nanoformulations. Although a few NG‐based formulations have been approved for clinical trials for subcutaneous delivery of antigen vaccines, the clinical translation of radiolabeled NGs for cancer imaging and therapy still remains a distant dream.^[^ ^31^, ^232^, ^233^, ^234^, ^235^, ^236^, ^237^ ^]^ Similar to other nanomedicines, the main drawback of NGs is that only 5–10% of the administered dose actually reaches the diseased site while maximum doses clear through the liver, spleen, and other organs.^[^ ^238^, ^239^ ^]^ The spleen is one of the responsible organs for foreign substrate filtration from blood. Generally, softer nanoparticles like NGs of size less than 200 nm can easily enter the spleen,^[^ ^240^ ^]^ whereas small nanoparticles (<5 nm) are cleared through renal filtration.^[^ ^241^ ^]^ Other than size, the shape of any nanoformulation also plays a crucial role in blood circulation time. For example, rod‐shaped nanoparticles circulate for longer time than the spherical ones.^[^ ^242^ ^]^ Opsonization of nanomedicine by MPS followed by clearance through liver and spleen is another issue for clinical translation.^[^ ^32^ ^]^ The surface charge of the NG can also influence the opsonization pattern as the NG having neutral surface charge demonstrates longer circulation time. Even so, the stimuli responsive behavior of the NGs are controlled by the charged groups present in the NG network. It is difficult to attain a balance between the charge related response of the NG and nonspecific interaction with other compounds present in vivo. Cationic polymer‐based NGs are commonly used for nucleic acid delivery.^[^ ^243^ ^]^ Complexation between cationic NGs and negatively charged DNA reduces the charge related in vivo toxicity. Therefore, shape, size, and surface charge of nanogels must be carefully controlled during the synthesis and radiolabeling procedures. Additionally, PEGylation, which is a commonly used method can be used to improve the circulation time and the biodistribution of the NGs and thus minimize the opsonization process.^[^ ^244^ ^]^

The probability of NGs to precisely deliver chemotherapeutic drug at tumor site is also limited even though their size is small enough as the structure of NGs might get deformed by interstitial fluid pressure.^[^ ^245^ ^]^ The vasculature in many hypoxic tumor is under developed and many large tumors tend to develop necrotic and hypoxic core which do not permit entry of NG via enhanced permeability and retention (EPR) effect, limiting passive targeting efficiency only to small tumors.^[^ ^32^ ^]^ Active targeting of NG using receptor‐targeting ligands might improve binding efficacy but this strategy also has its own limitations. It is difficult to find single receptor, which is exclusively overexpressed, only in desired tissue. For instance, folate receptor can be found in a large number of malignancies as well as in normal organs like kidney, placenta, and their homogeneous distribution can eventually lead to nonuniform accumulation of drugs.^[^ ^246^ ^]^ Moreover, the targeting ligands present in the NG network can affect its property like hydrophobicity, charge that may increase opsonization and rapid clearance by MPS. Apart from these, the high molecular weight of NG does not let them get removed from the body by kidney if they are nondegradable as the renal clearance threshold is ≈40 kDa. Hence, the biodegradable polymers are preferred but chemical functionalization may alter the polymer degradation rate. Therefore, detailed investigation about the metabolic profile of any NG along with the choice of suitable targeting ligand is essential before the proposal of clinical translation. It is challenging to control both the rate of drug release triggered by multiple stimuli and the degradation kinetics of NGs simultaneously. The burst release of the cargo from NGs can cause substantial loss of drug, which in turn might lead to little delivery to the site of interest and cause toxicity to the healthy organs. Nevertheless, these shortcomings can be circumvented by altering the polymer composition used for NG synthesis. Hence, an ideal NG‐based system should be able to balance all these factors to qualify for future clinical studies. The manufacturing process of NGs faces obstacles due to their complex chemistry, challenges of achieving reproducibility, and need to ascertain optimal physicochemical parameters.

Furthermore, the advancement is impeded by high cost in terms of material expenses and labor, shortcomings of regulatory guidelines, uncertainty of investor, and societal reluctance to accept new things, all contributing toward longer period for clinical translation of NGs.^[^ ^247^ ^]^ Current Good Manufacturing Practices (cGMP) guidelines must be followed before clinical transition of NG in order to guarantee their quality, safety, and efficacy for use in human trials. The production and quality control of radionuclides should also be performed in accordance with the cGMP guidelines. It cannot be denied that the overall field of nano‐oncology is complex, and the efficacy of any nanomaterial, including NGs, indeed depends on the specific application and formulation. Although, radiolabeled NGs signify a promising future for cancer theranostics and open new possibilities in the era of precision oncology, it is still in its infancy and yet to traverse a long distance before regular use in clinical context for the benefit of cancer patients. The long‐term stability of radiolabeled NGs needs to be evaluated in detail and this holds the key toward potential clinical translation. Despite several limitations, we remain optimistic about the potential of NGs as several other nanoparticle‐based therapeutic systems have already gained approval for clinical use.^[^ ^248^, ^249^, ^250^, ^251^, ^252^, ^253^, ^254^ ^]^

## 8 Conclusions

In this review, we have provided an overview about radiolabeled NGs for cancer diagnosis and therapy. Since the last two decades, applications of NGs have grown rapidly in biomedical field due to their biodegradable nature, high drug loading capacity, and stimuli‐controlled behavior for delivering drugs. Furthermore, progress in nuclear imaging techniques such as PET, SPECT along with other molecular imaging modalities made it possible for radiolabeled functionalized NGs to produce precise information about pharmacokinetics and biodistribution in vivo. Notably, multimodal imaging techniques involving radiolabeled NGs offer a comprehensive approach to understanding biological systems, particularly in cancer therapy. Unlike single modalities, multimodal imaging harnesses diverse modalities such as SPECT, PET, MRI, and optical imaging, providing complementary molecular and anatomical insights into living objects. NGs, with their adaptable structure, excel in cancer therapy by facilitating high drug loading capacities, while their flexibility enables efficient radiolabeling with therapeutic radionuclides, augmenting treatment efficacy. Furthermore, integrating radiolabeled NGs into combination therapy shows great potential for advancing treatment approaches by boosting therapeutic effectiveness through the synergistic action of therapeutic radionuclides and other treatment modalities. Although preclinical studies of radiolabeled NGs show promising results, optimizing their performance requires meticulous engineering to navigate the intricacies of biological systems. Rational design and refinement of NG properties are imperative to unlock their full potential, ensuring tailored solutions for personalized cancer treatment with enhanced therapeutic outcomes. The biggest hurdle of any NG is to overcome the biological barrier as due to their size they readily get opsonized from the system. Challenges also exist in terms of scalability, reproducibility, as well as targeted delivery and efficient clearance of the NG from biological system after accomplishing drug delivery. Therefore, concerted effort of all stakeholders, including, synthetic chemists, cancer biologists, radiochemists, and radiopharmacists should come to the forefront to take radiolabeled NGs from bench to bedside.

## Conflict of Interest

Weibo Cai declares conflict of interest with the following corporations: Portrai, Inc., rTR Technovation Corporation, Four Health Global Pharmaceuticals Inc. All other authors declare no conflict of interest.
